# The interplay of collagen, macrophages, and microcalcification in atherosclerotic plaque cap rupture mechanics

**DOI:** 10.1007/s00395-024-01033-5

**Published:** 2024-02-08

**Authors:** Imke Jansen, Rachel Cahalane, Ranmadusha Hengst, Ali Akyildiz, Eric Farrell, Frank Gijsen, Elena Aikawa, Kim van der Heiden, Tamar Wissing

**Affiliations:** 1 Department of Biomedical Engineering, Thorax Center Erasmus MC, University Medical Center Rotterdam, Rotterdam, The Netherlands; 2 Mechanobiology and Medical Device Research Group (MMDRG), Biomedical Engineering, College of Science and Engineering, University of Galway, Galway, Ireland; 3 Division of Cardiovascular Medicine, Department of Medicine, Center for Interdisciplinary Cardiovascular Sciences Brigham and Women’s Hospital, Harvard Medical School, Boston, MA USA; 4 Biomechanical Engineering, Technical University Delft, Delft, The Netherlands; 5 Department of Oral and Maxillofacial Surgery, Erasmus Medical Centre, Rotterdam, The Netherlands

**Keywords:** Atherosclerosis, Tissue mechanics, Collagen, Macrophages, Microcalcifications

## Abstract

The rupture of an atherosclerotic plaque cap overlying a lipid pool and/or necrotic core can lead to thrombotic cardiovascular events. In essence, the rupture of the plaque cap is a mechanical event, which occurs when the local stress exceeds the local tissue strength. However, due to inter- and intra-cap heterogeneity, the resulting ultimate cap strength varies, causing proper assessment of the plaque at risk of rupture to be lacking. Important players involved in tissue strength include the load-bearing collagenous matrix, macrophages, as major promoters of extracellular matrix degradation, and microcalcifications, deposits that can exacerbate local stress, increasing tissue propensity for rupture. This review summarizes the role of these components individually in tissue mechanics, along with the interplay between them. We argue that to be able to improve risk assessment, a better understanding of the effect of these individual components, as well as their reciprocal relationships on cap mechanics, is required. Finally, we discuss potential future steps, including a holistic multidisciplinary approach, multifactorial 3D in vitro model systems, and advancements in imaging techniques. The obtained knowledge will ultimately serve as input to help diagnose, prevent, and treat atherosclerotic cap rupture.

## Introduction—atherosclerotic plaque cap rupture risk

Failure of an atherosclerotic plaque is the culprit of many, often disabling or lethal, cardiovascular events, including myocardial infarction and stroke [15, 99, 144]. These acute events are associated with vascular biomechanics, and the cellular composition and matrix architecture of a plaque. The most common cause of failure of the atherosclerotic plaque is rupture, accounting for approximately 60% of events. Plaque erosion (35% of events) and calcified nodules (up to 5% of events) can also cause thrombi, however less frequently [249]. The probability of rupture is dependent on the stage of the atherosclerotic lesion. Virmani and colleagues proposed a lesion classification scheme, based on histopathological evaluation, starting with intimal thickening and progressing to intimal xanthoma, pathological intimal thickening, fibroatheroma and finally the vulnerable thin-cap fibroatheroma [268]. This last phenotype is considered as the main lesion with potential for rupture and is, therefore, named vulnerable plaque. These vulnerable plaques are characterized by a thin collagenous cap (i.e., the fibrous cap), which is often inflamed, calcified, and depleted from smooth muscle cells (SMCs) [17, 18, 81, 119, 252]. This cap is overlying a large lipid and/or necrotic core which is accompanied by inflammation and intraplaque hemorrhage [72]. Although great advancements in clinical imaging have been made to accurately image those features associated with vulnerability [58, 66], like cap thickness [108], the size of the lipid and/or necrotic core [181], inflammatory cells [85], calcifications [254], and intraplaque hemorrhages [51], phenotypic identification of the vulnerable phenotype is still found to have suboptimal predictive power for future clinical events [68, 176, 259]. Traditionally, a cap thickness of less than 65 μm is accepted to define a rupture-prone coronary plaque, while for carotid plaques, this threshold is set to 200 μm [36, 201]. However, clinical studies have shown that physical activity can trigger atherosclerotic plaque rupture, even in coronary plaques with a thickness of 70–140 μm [127]. Furthermore, carotid plaques have also been show to rupture at thicknesses up to 500 μm [201]. As such, guidelines to identify vulnerable plaques continue to be revised, with various phenotypic features of atherosclerotic plaques playing a role in creating a rupture-prone phenotype.

Years of extensive research have exposed various biochemical processes that precede and initiate plaque failure, as excellently reviewed by others [17, 20, 89, 152]. Rupture itself is a mechanical event that generally occurs at the cap when local stress levels exceed local tissue strength, which underlines the importance of understanding cap mechanics to prevent and foresee plaque failure [9, 204]. The stress distribution within the cap depends on the cap geometry, the mechanical properties of the cap constituents, and the external loading conditions (e.g., blood pressure). Nevertheless, due to the inter- and intra-cap heterogeneity, the stress distributions, as well as ultimate strength values, and failure mechanisms are substantially different. A wide range in ultimate tensile cap strength values have been reported (158 kPa [234] to 870 kPa [111]). Importantly, geometry, composition, and resulting cap stress and strength will change in a spatiotemporal manner due to the iterative interplay between the cellular populations and environmental cues, including matrix properties and mechanical loading [96, 195, 207, 260].

To improve risk assessment, it is a requisite to highlight the importance of cap mechanics in plaque biology and cap failure. This review would like to create awareness for this topic by evaluating current knowledge on the role of cap constituents in plaque cap mechanical (de)stabilization. We hereby focus on the end-stage, vulnerable thin cap, and assess the role of three components present in this phase. The first constituent included is collagen, which is the main load-bearing component of the cap [101] and often scarcely renewed in vulnerable caps due to SMC depletion and potential senescence [17, 18, 81, 252]. Second, macrophages are discussed as major cell population correlated with an enhanced rupture risk [225] by directly initiating extracellular matrix (ECM) degradation via the secretion of degrading compounds like MMPs [175]. Third, the role of microcalcifications is emphasized, as being the most abundant type of calcification present in the vulnerable plaque cap [116]. Microcalcifications are included in this review due to their increasingly named role as local stress concentrators, possibly being a cause of cap rupture [42, 52, 116, 257]. We will elaborate on the contribution of these three constituents to cap mechanics directly, their biological interplay and discuss future work that could enhance our biomechanical understanding to better identify the cap at risk of rupture.

## Cap components: macrophages, collagen, and microcalcification

### Macrophages

Atherosclerosis is a lipoprotein-induced chronic inflammatory disorder [263]. Upon endothelial dysfunction, low-density lipoprotein (LDL) accumulate within the intima and oxidize, after which monocytes are recruited [49, 62, 147]. Inside the vessel intima, monocytes mature into macrophages, behaving as either more pro-inflammatory (i.e., M1) or anti-inflammatory (i.e., M2), depending on their activated intracellular signaling pathways and the cellular microenvironment [26, 231]. Although lesional macrophages are heterogeneous and can differentiate into various phenotypes, it is generally accepted that the M1/M2 ratio is high in vulnerable caps [76], with more pro-inflammatory-like macrophages in the shoulder regions and lipid core, while fibrous mid-cap sections generally contain both phenotypes [76, 228]. Many of the macrophages within the plaque can phagocyte lipids [146] and remove apoptotic cells and other cellular debris [217]. However, due to their inability to digest oxidized LDL, these macrophages become oversaturated with lipids and transform into foam cells, contributing to formation of a lipid pool and/or necrotic core [143].

In line with plaque heterogeneity, the macrophages that reside in the plaque are considered to be highly versatile and plastic [24, 54, 110, 149]. The generally proposed M1/M2 macrophage diversity is oversimplified, and various other phenotypes have been proposed to exist (i.e., M2a, M2b, and M2c). Recently, athero-specific subsets (M4, M(Hb), and Mhem) were observed in human plaques [24, 32, 54, 110, 149, 228, 262], but although they are considered to be either proatherogenic (M4) or atheroprotective ((M(Hb) and Mhem) [26], how these subsets affect their nearby microenvironment and alter cap mechanical properties is still largely unknown.

Of note, besides macrophages, various other immune cells play a role in the initiation and progression of atherosclerosis. In thin fibrous caps specifically, large numbers of macrophages and T cells were found to correlate with an enhanced rupture risk [225]. Herein macrophages are known as the principal cell actively secreting degrading compounds, including matrix metalloproteinases (MMPs) [182], whereas T cells predominantly have an instructing role stimulating macrophages to release MMPs [174]. Therefore, the macrophage is chosen as the focus of this review.

### Collagen

Soluble factors produced by inflammatory cells that reside within the plaque induce the migration and proliferation of vascular smooth muscle cells (SMCs) toward the intima. It is generally assumed that they undergo a transition from a quiescent, contractile phenotype to a proliferative, synthetic phenotype. SMCs synthesize ECM molecules, including collagen, elastin, as well as glycosaminoglycans (GAGs) and proteoglycans. These ECM components induce thickening of the intima layer and contribute to the formation of a fibrous plaque cap [144]. The composition of ECM components changes during atherogenesis. In advanced plaques, collagen is the major constituent of the ECM accounting for up to 60% of the total protein content [14, 82, 114, 203, 218], while in healthy arteries, the majority of protein is elastin [82]. Moreover, the ECM of fibrous caps of advanced plaques is subjected to aging as a consequence of reduced SMC proliferation and increased SMC death and senescence, while the growing core extends outwards, resulting in further thinning of the fibrous cap [17, 18, 81, 252]. The main collagen types in the fibrous cap of advanced plaques are the load-bearing fibrillar collagens type I and III [14, 114, 193, 218]. Both types are diffusely (co)distributed in plaques, with their local content and relative proportions varying both within and between plaques [114, 165]. The tensile strength and torsional stability of these collagens primarily regulate cap structural integrity [14, 114, 218]. Other collagen types regularly detected in atherosclerotic lesions include collagen type IV, V, VI, and VIII [114, 193, 250], with varying primary locations and functions.

Intermolecular covalent cross-links are formed between the collagen molecules, catalyzed by enzymes such as lysyl oxidase (LOX) [151, 161], and proteoglycan-rich matrix is deposited between fibrils that together affect collagen mechanical performance [230]. Furthermore, collagens in atherosclerotic plaques are exposed to extracellular glycation- or oxidation-induced cross-linking, also called advanced glycation end products (AGEs)-related cross-linking, impairing functional interaction of collagen with cells and stiffening the matrix [87, 210, 242]. AGEs in human plaques have been found predominantly in inflammatory atheromatous lesions that are often classified as rupture-prone plaques [92].

### Microcalcification

It is currently widely accepted that vascular calcification is an active, cell-mediated process [40, 139], instead of a passive accumulation of calcium. Crystalline hydroxyapatite (HAP) is the main component of calcifications [166, 171]. In addition, precursors of HAP, such as amorphous calcium phosphate, octacalcium phosphate, and dicalcium phosphate dehydrate, have been reported in atherosclerotic lesions [88, 133, 191, 205, 270].

Multiple mechanisms for the formation of calcification have been suggested. One of these is the release of SMC- or macrophage-derived [115] calcifying extracellular vesicles (EVs), where calcium crystals nucleate on the EV surface and mature over time into HAP [105, 191]. Another potential pathway is osteochondrogenic trans-differentiation of vascular SMCs. Several lines of evidence showed that atherosclerotic calcification shares features with bone formation [148, 183, 219]. Apoptosis of vascular SMCs and macrophages also seems to play a substantial role in the onset of calcification, as calcium deposits are often located near or in the necrotic core and in close proximity to apoptotic cells [110, 164]. Moreover, loss of calcification inhibitors is known to influence calcification formation. All these mechanisms have been extensively reviewed [31, 40, 124, 134, 183, 209].

Different categories of calcifications can be observed in the cap, categorized by their size. Macrocalcifications can be divided into three types: speckled, spotty calcifications (~ 50 µm), sheet-like fragments (> 2 mm) and diffuse segments of calcifications (> 5 mm) [15]. Various studies have shown that macrocalcifications can lower overall stresses in the cap, due to their load-bearing capacity [150, 264]. Where macrocalcifications are, thus, often seen as a cap-stabilizing component, computational studies show that microcalcifications (< 50 µm) might act as local stress concentrators. Microcalcifications are distributed heterogeneously throughout the necrotic core, where they are believed to be not mechanically relevant, and in the cap [156], where they can contribute to rupture [42, 52, 116, 257]. Since it has been shown with high-resolution µCT that microcalcifications are the most abundant type of calcification in the vulnerable plaque cap [116], we have chosen to focus on the role of microcalcifications on cap mechanics in this review.

## Cap components: role in cap mechanics

**Fig. 1:**
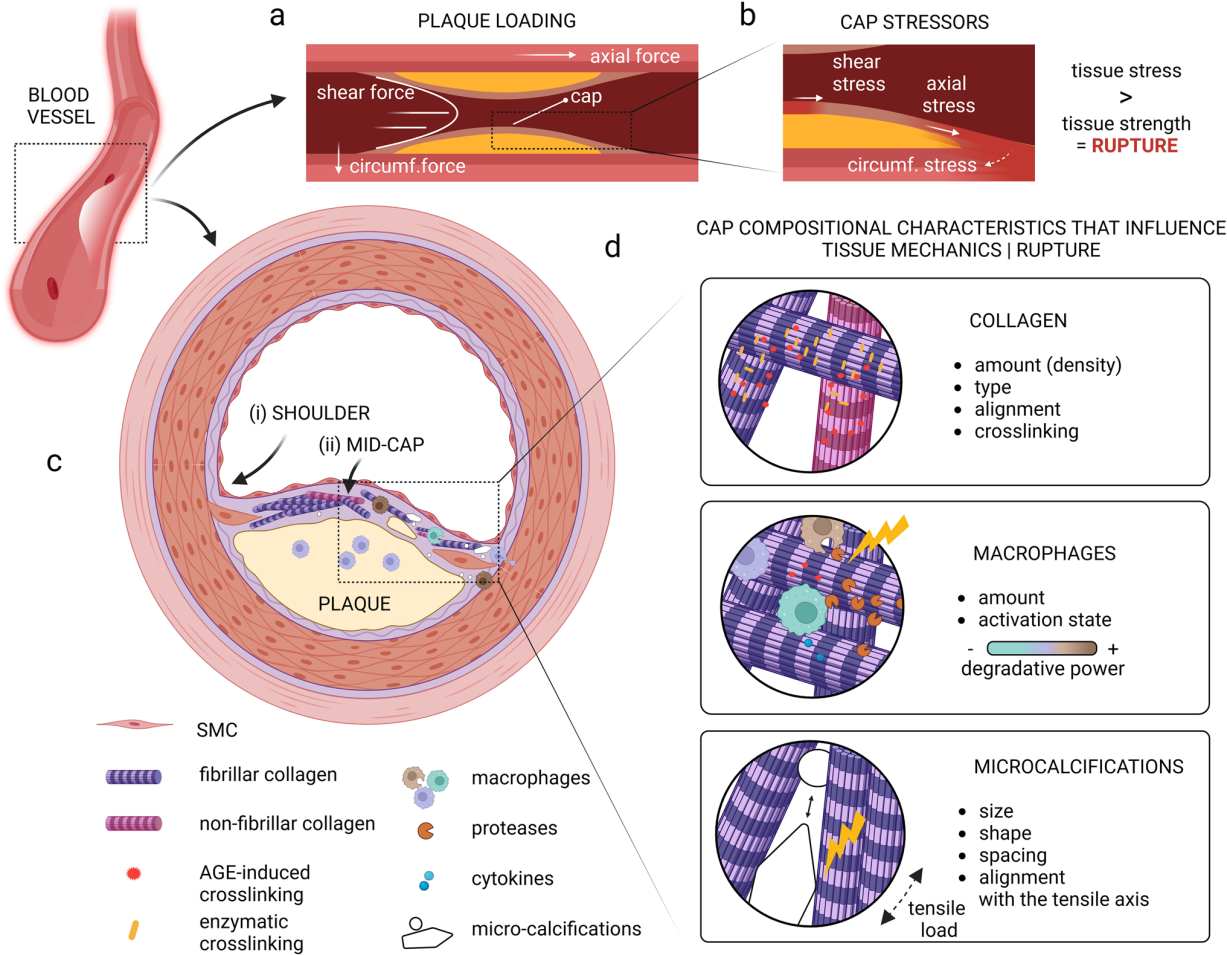
Graphical representation of a vulnerable plaque and cap with its most important mechanical stressors, cap components, and their influence on tissue mechanics. **A** A simplified graphical representation of a longitudinal section of an atherosclerotic plaque with the primary forces acting on it. **B** Zoom-in of the cap region with the primary stressors locally initiating tissue stress. **C** Graphic of a transverse section of a vulnerable plaque with a fibrous cap, showing the shoulder (i) and mid-cap (ii) regions. **D** The cap compositional characteristics which influence tissue mechanics, being collagen, macrophages, and microcalcifications, and their effect through various individual characteristics on cap mechanics and consequent cap rupture. Created with BioRender.com

Where the impact of mechanics is extensively studied in atherogenesis [95], its role in cap rupture is evident but less well understood. Plaques are exposed to various mechanical forces (Fig. 1A) that can induce peculiar mechanical behaviors that vary with plaque geometry, location, and composition [9, 101]. Thin caps of vulnerable plaques must possess sufficient strength to endure the force-driven stresses to which they are exposed (Fig. 1B). The most influential stress experienced by the cap is the circumferential stress induced by the blood pressure, acting perpendicular to the arterial wall, leading to deformations within the cap. The effect of blood-flow-induced shear stress on cap deformation is believed to not be substantial as circumferential stresses are orders of magnitude higher. Their influence on cap rupture can, however, not be neglected as shear stress is believed to affect cap erosion as well as endothelial function and thereby cap biological composition [84, 160]. Axial tensile stress, as a consequence of among others hemodynamic loading, differences in vessel geometry and plaque morphology, is believed to predominantly affect the rupture events upstream of the plaque where these stresses are highest [223].

As cap constituent material properties affect the stress distribution throughout the cap and determine the strength of the cap, this section specifically elaborates on the variations in collagenous matrix properties, macrophages, and microcalcification seen within human atherosclerotic plaque caps (Fig. 1C) and the impact thereof on cap mechanics and stability (Fig. 1D). Cap mechanics and stability are hereby described on the mesoscale by terms such as tissue stiffness and strength as defined in Table 1. For an in-depth description of the complexity of plaque biomechanics, nano- and micro-scale mechanics of the collagenous matrix, as well as the effect of collagenous matrix properties on SMC-driven matrix formation and degradation, we refer the reader to the following literature [9, 39, 80, 84, 193, 203, 223, 255, 265].

**Table 1:** Mechanics terminology

Descriptor	Description*
Strength	The maximum capacity to withstand a load, expressed as a stress threshold value
Ultimate tensile strength/stress	The maximum stress that can be withstand while being exposed to tensile loads
Stress	Force per unit area
Peak circumferential stress	The highest stress value in the circumferential direction (the direction that undergoes (highly likely) the largest deformation in vivo)
Strain	Deformation measure
Uniaxial extension	Stretch in only one direction
Stiffness	Measure of the material’s resistance to elastic deformation
Elasticity	The ability to return to its original shape after undergoing deformation
Elastic (Young’s) modulus	Measure of the material’s resistance to elastic deformation, assuming a linear response
Tensile testing	A test wherein a sample is subjected to a controlled tensile load, often until failure

*Various definitions possible, however aimed at simple definitions for non-expert in the field

### Role of collagen on cap mechanics

The plaque cap resistance to rupture largely depends on the load-bearing collagenous network [8, 100, 103]. This aspect in turn depends upon the balance between collagen synthesis and degradation, the latter being significantly affected by macrophages that secrete proteases [145] as discussed in “Interplay between macrophages and collagen”. Although increasing collagen amount has been correlated with increments in stiffness and strength for human plaques and their fibrous caps [43, 59, 135], correlations were not always convincing. This can be explained by the knowledge that not solely amount but the cumulative effect of nano- to macro-scale collagen properties determine its mechanical behavior [80]. Variations in molecular structure and intracellular and extracellular processing can reflect in the collagen type, alignment as well as its cross-linking. This section focuses on those studies that explored the effect of these properties on cap mechanics.

The primary contributors to the mechanical strength of tissue in the cap are the fibrillar collagens [14, 230]. Since collagen type I is believed to be profibrotic and modulate tensile strength, and collagen type III is associated with tissue elasticity [193, 222], the relative presence of both types is expected to affect mesoscale cap mechanical behavior. To assess this impact, Burleigh et al. compared the collagen type I and III proportion with the ultimate tensile strength for ulcerated and non-ulcerated human aortic plaque caps and adjacent intima. Where the strength of the collagen in the caps appeared to be lower when compared to the collagen of adjacent intima, the proportion of collagen type I to III was not related to the ultimate tensile strength [37]. Although not directly related to atherosclerosis, several studies in tendons did demonstrate an impact of varying proportions of collagen type on tissue stiffness. These studies showed that the quantity of collagen type III is inversely correlated to the elastic modulus [12, 34]. These variances in tissue stiffness are believed to be related to the differences in protein structure, and fiber and fibril diameter [3, 23]. The presence of fibrillar collagen type V, as well as the non-fibrillar collagen types IV, VI, and VIII in atherosclerotic plaques potentially influences the tissue mechanical stiffness and strength via affecting either collagen type I fiber diameter (type V) [3], SMC activation (type IV) [14] or the interaction with other ECM components (type VI/VIII) [21, 193], though their direct effect on plaque tissue mechanics remains to be explored.

In addition to collagen type, several studies have demonstrated that less dispersed alignment of collagen fibers into the load direction correlates with greater tissue strength and/or stiffness for both healthy arteries [214] as well as atherosclerotic plaque caps [111]. However, where collagen fibers in healthy arteries have a distinct oriented collagen network, intra- and inter-plaque fiber orientation vary substantially [8]. Within human coronary plaques, shoulder regions (Fig. 1A) were defined by Douglas et al. as more dispersed and less aligned when compared to the mid-cap [64], while Tornifoglio et al. showed that the rate of disorganization and microstructural arrangements varied considerably between human plaque caps [238]. Mechanically, the more disorganized samples appeared to be weaker in comparison to the samples with a more predominant circumferential orientation, when exposed to uniaxial tensile testing in the circumferential direction [238]. These findings suggest that collagen fiber alignment might be a relevant parameter for rupture risk assessment.

Finally, cross-linking of collagen is known to impact local and global mechanical properties. Enzymatic cross-linking of collagen, a process controlled by enzymes such as LOX, is believed to be essential for collagen fibril structure and integrity, which directly influences the tissue strength [198, 224]. In human plaques, LOX presence was associated with a more stable plaque phenotype and LOX expression correlated negatively with markers of immune activation and the incidence of myocardial infarction (MI) [185], suggesting a protective mechanism. Mechanically, LOX deficiency has been shown to cause serious collagen network weakening [154] and, although related to human heart valves, enzymatic cross-links were shown to play a dominant role over collagen content affecting valve biomechanical tissue behavior [13].

As the cross-linking of collagen reduces its sensitivity to proteolytic degradation [159], one could suggest that cross-linking enhances cap mechanical strength. However, a reduced sensitivity to proteolytic degradation also contributes to impaired matrix remodeling, as tissues with a slow turnover are more susceptible for irreversible AGE-driven cross-linking [79]. AGEs are related to enhanced tissue stiffness [69, 224], due to limited sliding between fibers and fibrils [79].

In conclusion, it is the sum of all collagen properties, such as type, orientation, and cross-linking, that determines its mechanical strength and stiffness.

### Role of macrophages on cap mechanics

Macrophages play an important role in the onset and progression of atherosclerosis, as well as the process of plaque cap rupture [11]. More pro-inflammatory macrophages are present in the shoulder region [228], where 65% of ruptures occur [153]. One of the first studies which demonstrated that caps of ruptured plaques have higher amounts of macrophages compared with intact caps was performed by Lendon et al. [136]. In this study, strips of plaque cap tissue underwent uniaxial extension and ruptured caps were compared to intact caps. Higher macrophage accumulation was associated with a lower ultimate tensile stress [136], supporting the hypothesis that macrophage infiltration increases rupture risk. By combining immunostaining of macrophages and the onset of events in patients, a positive correlation was found between macrophage infiltration and the occurrence of cerebral ischemic events [104] and acute coronary symptoms [163]. Similarly, the analysis of cellular characteristics in rupture sites of thrombosed arteries extracted from MI patients revealed macrophages to be the dominant cell type at the site of rupture, suggesting an active inflammatory reaction [251]. Kolodgie et al. demonstrated extensive apoptosis of macrophages at the rupture site by staining lesions of sudden coronary death, suggesting a potential destabilizing role of apoptotic macrophages in plaque caps [120]. However, it should be noted that analysis of caps post-rupture cannot be used to conclude that macrophages were the primary cause of rupture.

To be able to assess the role of macrophages on local tissue mechanical properties, intravascular elastography was performed [122, 212], and local high-strain spots were correlated with the presence of macrophages [122]. Uniaxial tensile tests with notched fibrous cap tissues were performed to characterize rupture behavior, where caps with higher macrophage density were found to rupture at lower stresses [59]. Potential correlations between macrophage presence and rupture behavior could be explained by the indirect role that macrophages play in the degradation of the matrix, which will be elaborated on in “Interplay between macrophages and collagen”.

The experimental results discussed above were validated by computational modeling using a patient-specific set of 3D computational models [232]. To be able to reflect inflammation in the model, material stiffness in the cap was reduced to reflect cap weakening due to inflammatory processes. This weakening led to large cap strain conditions when combined with a thin cap and hypertension. Furthermore, the lower stiffness led to lower cap stress [232].

Macrophages and inflammatory processes, thus, seem to affect cap mechanics and rupture. However, the number of experimental studies is limited.

### Role of microcalcification on cap mechanics

Microcalcifications are prevalent in atherosclerotic caps and non-uniformly distributed in the shoulder and mid-cap regions [157]. These calcified deposits are believed, based on ex vivo histopathological observations combined with computational simulations, to influence cap rupture [204].

Microcalcifications can amplify the stresses in the cap [25, 41, 200, 245, 246] and transfer the peak cap stress (PCS) to their location in the cap [257]. Moreover, microcalcifications have the most detrimental effect depending on their location [257]. Specifically in regions of high stress [245] or thin caps [245, 246], microcalcifications can induce high mechanical stress to promote cap rupture.

Aside from location, morphological characteristics of the microcalcification, such as the size and shape, can influence the fibrous cap stress. A critical microcalcification diameter is described by Kelly-Arnold et al. from 5 to 65 μm. Outside this range, microcalcifications are thought to be less harmful [116, 155]. This critical size is, however, also dependent on their localization, as microcalcifications located in a thin cap were shown to compromise the mechanical stability of the cap the most [56]. It is currently, however, not known in what way the ratio of the microcalcification size and cap thickness influence each other. Distinct from the spherical microcalcifications, elongated microcalcifications substantially increase the cap stress [41, 52, 245]. In addition, higher volume fraction of microcalcifications also increases the fibrous cap stresses [257], and interparticle spacing has been shown to be a principal determinant of rupture risk [155]. Cardoso et al. showed that local stress levels are increased by a factor of five for closely spaced microcalcifications [41, 116, 157]. In the fibrous cap, most microcalcifications are situated together in clusters [109], which could, thus, increase the stress accumulation even more than a single microcalcification. Furthermore, their alignment with the tensile axis can significantly increase cap stress [41, 52, 116]. Notably, these findings are almost all based on numerical studies.

Experimentally, the effect of micro-beads, representing microcalcifications with varying diameters and concentrations, within a silicone-based material has been investigated [55]. Larger beads (diameter > 80 μm) reduced the ultimate tensile stress significantly, while smaller beads (diameter 6 μm) only were of effect in thin (100 μm) experimental samples. Furthermore, higher concentrations of particles correlated with lower stresses at rupture[55], endorsing the results from numerical models.

Microcalcifications are, thus, assumed to act as local stress amplifiers in the fibrous atherosclerotic cap, with microcalcification size, shape, spacing, and alignment with the tensile axis as key determinants in the resulting stress.

## The interplay between collagen, macrophages, and microcalcification

**Fig. 2:**
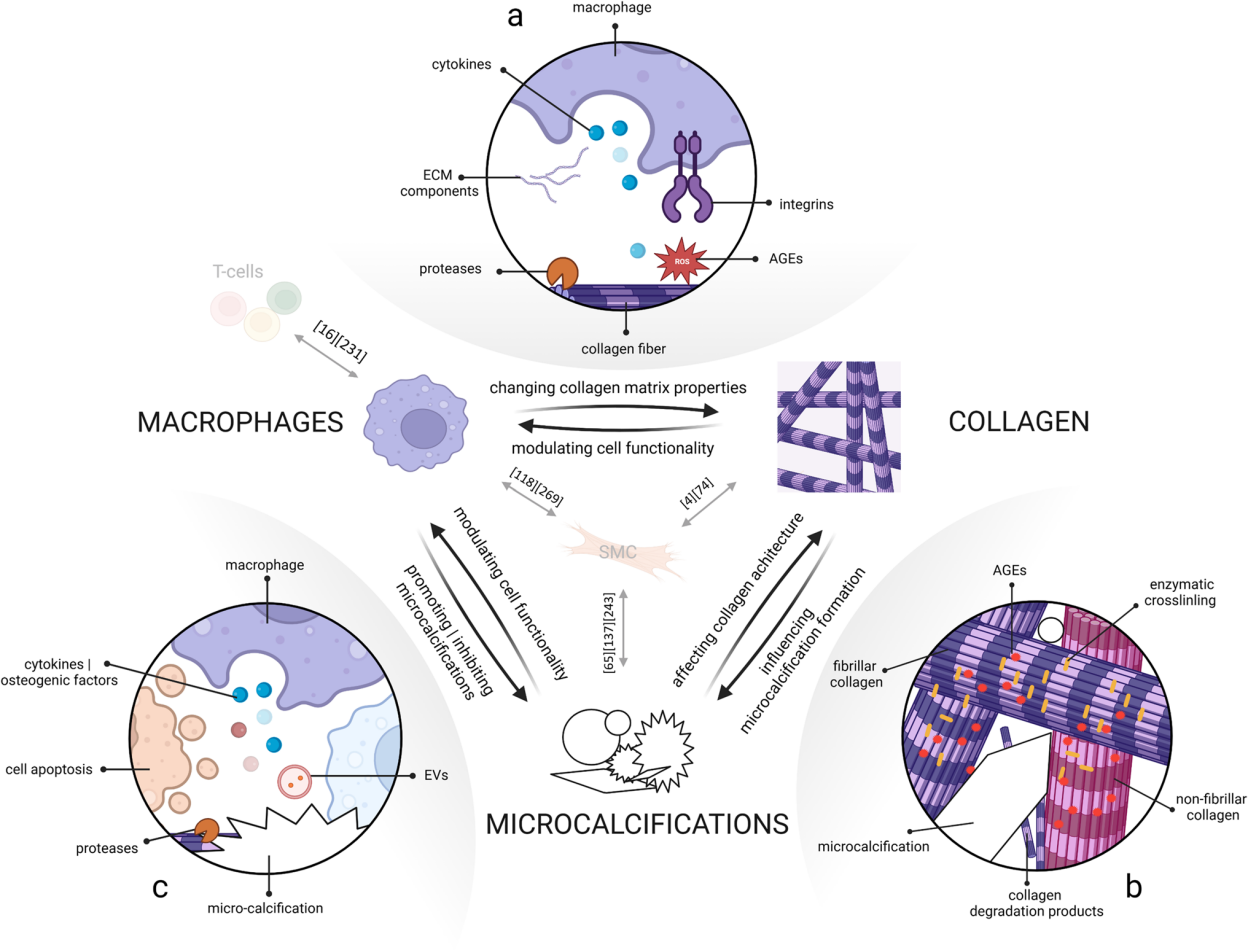
The interplay between the main cap components: collagen, macrophages, and microcalcifications and essential reviews discussing the influence of SMC’s and T cells on these components [16, 65, 74, 118, 137, 231, 243, 269].  **A** Macrophages influence the collagenous matrix by secretion of cytokines, ECM components, AGEs, and proteases. Furthermore, integrins detect matrix properties and regulate processes of macrophage biology, by which the collagenous matrix properties, thus, modulate cell functionality. **B** Collagen serves as a scaffold for microcalcification formation and collagen type, cross-linking and degradation can affect the formation of microcalcifications. Furthermore, microcalcifications themselves affect collagen architecture. **C** Macrophages release cytokines, osteogenic factors, proteases, and EVs that can alter calcification formation. In addition, macrophage apoptosis contributes to microcalcification formation. Microcalcifications, in turn, modulate macrophage functionality by promoting release of pro- or anti-inflammatory factors. Created with BioRender.com

While collagen, macrophages, and microcalcifications each distinctively affect cap mechanics, as discussed in “Cap components: role in cap mechanics”, they cannot be considered as independent contributors. Their continuous interplay affects plaque cap development and ultimate rupture risk (Fig. 2). This section sets forth on the current knowledge regarding their interplay. Other cellular players that are generally accepted to be involved in fibrous cap destabilization, like T cells and SMCs, are occasionally mentioned. Figure 2 provides an overview of the most important reviews describing their interplay with the three compositional parameters elaborated on in this review.

### Interplay between macrophages and collagen

Regarding collagen-dependent cap strength, macrophages play a dual role, presenting a matrix-degrading or a matrix-preserving role. Each role is accompanied by the secretion of a specific set of active compounds that cumulatively affect cap (in)stability. This section focusses on how macrophages degrade or preserve their surrounding collagens, by secreting (i) proteases, (ii) cytokines, (iii) ROS, and (iv) ECM components, and ends with the reciprocal effect of the collagenous matrix itself on macrophage biology.

The most underlined contribution of the macrophage to plaque rupture involves the secretion of proteases, including metalloproteases (MMPs), cysteine (e.g., cathepsins), and serine proteases [63, 126, 175, 247]. There is compelling evidence that human atherosclerotic plaques are associated with the presence of macrophages and proteases, and this presence was linked to thin fibrous caps, and even cap rupture [1, 63, 229]. While an excess of degrading proteolytic enzymes over their inhibitors contributes to collagen remodeling and cap destabilization [175, 266], it is worth noting that the type and amount of protease and their inhibitors secreted by macrophages can vary based on their differentiation state and the stimuli they perceive [175, 186].

The load-bearing fibrillar collagens type I and III are resistant to most proteases and can only be cleaved by certain MMP collagenases, including MMP-1, -8, -13, and -14 (MT1-MMP) [70, 71] that can be secreted by macrophages in copious amounts [173, 175]. These collagenases cleave collagen I–III into fragments that quickly denature into gelatin, which can be further degraded by MMP-2 and -9 [4].

Although MMP-2 and -9 are classified as gelatinases, they also possess collagenolytic activities that affect fibrillar collagens [7, 22, 189]. Of the cathepsin family, cathepsin K has been identified as the most potent cathepsin affecting tissue strength, by cleaving proteoglycan-GAG interfibrillar bridges as well as fibrillar collagens [73, 142, 187].

Of note, proteases not only actively degrade collagen; MMP-2, -4, and -9 are known stimulators of SMC proliferation and migration [172], suggesting a possible matrix-preserving and stimulatory role for these macrophage-secreted proteases.

Besides proteases, macrophages secrete various active compounds. Frequently observed ones to be present in vulnerable atherosclerotic plaques include the inflammatory cytokines IFN-ϒ [30], TNF-α [188], as well as ROS [179] that all have been described to increase protease secretion and activity [199, 229, 266], contributing to collagen degradation. Moreover, they have been shown to also destabilize the collagenous matrix by either being involved in SMC apoptosis, inhibiting SMC proliferation, reducing collagen production or inhibiting LOX expression in vascular human or animal SMCs [10, 33, 93, 184, 226], increasing plaque vulnerability.

Cytokines that are generally thought to be on the matrix-preserving side include platelet-derived growth factor (PDGF) and transforming growth factor-β (TGFβ). PDGF has been found within macrophages in all phases of atherosclerotic plaque development [206] and is known for its mitogenic potential stimulating SMC migration and proliferation, inducing intimal growth [159]. TGF-β is secreted by the more anti-inflammatory activated macrophages [26] and has a well-established role in ECM synthesis, enhancing collagen deposition [208, 237]. While both factors have been correlated to tissue stabilization, their protective role regarding cap rupture must be interpreted with caution as some studies show correlations to cellular apoptosis [202], calcification [220], and cap rupture [28].

Macrophages are furthermore a source of AGEs, that are formed in conditions of hyperglycemia or oxidative stress, which are seen in aging, diabetes, and settings of inflammation and hypoxia [242]. AGEs have been shown to promote vascular damage and accelerate atherosclerotic plaque progression, partly through a direct mechanism of altering ECM molecules, including collagen. AGE products are known to form protein adducts or cross-links, and accumulate for years, contributing to an irreversible, highly cross-linked collagenous matrix and a stiff fibrous cap [79, 121, 242]. It was discovered that macrophages can synthesize ECM components, including collagen, themselves [123, 215, 256]. Studies showed that macrophages express all collagens and collagen-related mRNAs and were able to secrete collagen type VI and VIII considerably in vitro [215, 256] and in human atherosclerotic plaque conditions [256].

Finally, the collagenous matrix itself plays an important role in regulating various processes of macrophage biology, including monocyte-to-macrophage differentiation, lipid uptake [258], metabolism [244], polarization [131], migration mode [86] as well as their ability to secrete proteases [138, 258]. It has been shown in vitro that macrophages increase their MMP-9 secretion when cultured on monomeric type I collagen, in comparison to when they were exposed to the polymerized variant [138]. Lastly, AGEs are known to affect cells via its interaction with the key receptor for AGE (RAGE), which is present in almost all cells that reside in a plaque, including macrophages, and induces the subsequent secretion of pro-inflammatory compounds, including ROS [242].

In conclusion, macrophages secrete a plethora of multifaceted active compounds that cumulatively determine collagenous matrix properties and rupture risk. In turn, macrophage biology is affected by collagen. Therefore, macrophage and collagen continuous crosstalk can contribute to changes in the local microenvironment that promote plaque rupture.

### Interplay between collagen and microcalcification

Collagen plays a complex and multifactorial role in microcalcification formation, and many of the underlying mechanisms are currently being studied. Collagen characteristics that are named to influence microcalcification formation include (i) amount, (ii) integrity, (iii) type, (iv) alignment, and (v) cross-linking.

Hutcheson et al. found an inverse relationship between collagen amount and calcification, with microcalcifications forming in regions of collagen fiber degradation [105], as disrupted or fragmented collagen fibers may serve as nucleation points for microcalcification formation [5]. By aggregation of EVs in between the collagen fibers, calcifying structures were formed [105]. Collagen was shown to act as a scaffold for microcalcification formation, where collagen alignment directly influences microcalcification shape, by modulating calcification EV aggregation to gain an elongated morphology [105]. Thus, local collagen organization can influence calcification development and create a multitude of calcification-fibrous tissue interactions, with varying effects on tissue mechanics [15]. Calcifying EVs locate in proximity to collagen fibrils [113, 213] and interestingly, EVs isolated from vascular SMCs were only capable of inducing calcification along collagen type I fibrils [47]. Type I collagen was also shown to promote calcification of vascular SMCs, by stimulating their differentiation into osteoblast-like cells [102, 112, 177] or increasing mineralization parameters such as calcium incorporation and mineral formation [255]. Type IV collagen, on the contrary, inhibited these processes [255]. The overall matrix stiffness is also a known contributor to vascular calcification [177]. Matrix stiffness can be detected by matrix-binding cell surface receptors such as discoidin domain receptor tyrosine kinases (DDRs) and integrins, present on vascular SMC [4]. DDR-1 has been shown to act as a mechanical sensor altering matrix deposition [125, 255], activating MMPs [29], promoting the secretion of EVs [125, 255] and regulating osteogenic differentiation of SMC [178], ultimate processes that contribute to calcification. Matrix stiffening, as a result of AGE-dependent cross-linking, was found to hinder collagenase-mediated degradation, subsequently promoting SMC differentiation into adapting the osteogenic phenotype [235]. In addition, LOX-dependent cross-linking was linked to vascular calcification [112].

Taken together, various collagen characteristics affect microcalcification formation, but there is a paucity of information on reciprocal effect of microcalcifications on collagen properties. In view of tissue mechanics, one could speculate that microcalcifications can affect collagen remodeling processes, while microcalcification-induced stress accumulations might locally affect collagen integrity. Furthermore, microcalcifications can merge to form macrocalcifications, causing altered interactions with the collagenous matrix. Advanced macrocalcifications can be also formed when collagen fibers themselves calcify [15, 98], but osteoid metaplasia resembling bone structure is relatively rare event. Gijsen et al. showed that four distinct fiber patterns around macrocalcifications can be recognized: attached, pushed-aside, encircling, and random [83]. Only in the first pattern, the fiber structure was still visible inside the calcifications, indicating that this type of calcification might develop due to fiber mineralization, while the others are formed due to the agglomeration of EVs in between the collagen fibers [83].

### Interplay between macrophages and microcalcification

The hypothesis that macrophages and microcalcifications are involved in a complex interaction first originated from studies where arterial inflammation was found to be correlated with the localization of calcifications [2, 35, 44, 169]. Microcalcifications were shown to induce M1 macrophage polarization [162], in comparison to macrocalcification, that was associated to a M2 phenotype instead [130, 132, 227].

The reciprocal effect of inflammation on microcalcification formation has been described at various interconnected levels: (i) the release of calcifying EVs; (ii) through the production of proteases; (iii) through the release of cytokines and osteogenic factors; and finally (iv) through the induction of apoptosis.

First, the release of calcifying EVs plays a role in the formation of microcalcifications. Cells present in the atherosclerotic cap, such as vascular SMCs and macrophages, secrete EVs that are loaded with mineralization inducing factors, including tissue non-specific alkaline phosphatase (TNAP), thus serving as a nucleating point for calcification [105, 106]. Hutcheson et al. demonstrated that aggregation of multiple calcifying EVs causes the formation of microcalcifications in atherosclerotic lesions [105]. In addition, it has been demonstrated that macrophage-derived EVs, enriched with S100A9 and Annexin V, accelerate microcalcification formation in chronic kidney disease and diabetes mellitus [115, 171]. Taken together, vascular cell-derived EVs can be seen as active contributors and building blocks of microcalcifications.

The second pathway by which macrophages affect calcification is through collagen degradation, as discussed above, resulting in nucleation loci for calcification [5, 141, 170]. Various MMPs, mainly produced by activated macrophages, have been associated with calcification. MMP-9 and MMP-10 enhance both inflammatory and calcifying processes [48, 197], and MMP-1 levels are found to be higher in calcified plaques compared to non-calcified lesions [194].

Besides these proteases, there are also factors released by macrophages such as cytokine TNF-α, that trigger osteoblastic activity in vascular SMCs, consequently promoting calcification [6, 107, 219]. Not only do macrophages trigger osteoblastic activity in vascular SMCs, they also release osteogenic factors directly. In the tumor microenvironment, macrophages have been shown to tune microcalcification formation by the release of osteogenic factors (e.g., BMP-2 [253]) and inhibiting factors (e.g., osteopontin [50, 180]). In line with the secretion of osteogenic factors, several studies suggested that macrophages themselves can contribute to an osteoblast-like or osteoclast-like phenotype, aggravating or resorbing vascular calcification [38, 75, 140].

Apoptosis of macrophages (and macrophage-induced apoptosis of SMCs [33]) might be yet another pathway resulting in the onset of calcification. Various lines of research have suggested that calcification initiates within cell debris, and that necrosis and apoptosis might, therefore, induce calcification by serving as nucleation sites [53, 67, 196]. Microcalcifications often form around the necrotic core [209], within a dense population of macrophages and apoptotic debris [120]. When phagocytic clearance of apoptotic bodies (i.e., efferocytosis) is compromised, apoptosis will transition into secondary necrosis, increasing the amount of calcification [236].

Not only do inflammatory processes affect calcification, but vice versa in a procalcified environment with elevated phosphate levels, macrophages shift to a M2 phenotype [248]. Within this in vitro study, macrophages showed anti-calcifying activity, which may protect the tissue from further calcification [248]. M2 macrophages phagocytize necrotic fragments and apoptotic cells [117], limiting nucleation sites for calcification. Macrophages also encapsulate, internalize, and resorb deposits of calcium [129, 168, 190] such as HAP particles or related precursors that have been shown to distinctly induce pro- [46, 168, 190], anti- [267] or ‘hybrid’ macrophage phenotypes in vitro, a process that depends on microcalcification size and shape [132, 167, 227]. Whether these observations can be translated to the plaque itself remain to be examined. Deep phenotyping studies of plaque macrophages in proximity to calcification would help explaining the interaction between calcification and macrophages. Resent histological evaluation of human carotid plaques showed that CD163 and CD86 positive cells correlated with macrocalcifications, while plaques with microcalcifications had a high presence of M1 macrophages [162]. On the contrary, another study showed that CD163 + M(Hb) macrophages can restrain vascular calcification in vitro and demonstrated an inverse correlation between CD163 + macrophages and vascular calcification in human atherosclerotic plaques [211].

Overall, these observations insinuate that microcalcifications are not merely a passive result of persistent inflammation but also initiate a feed-back loop controlling inflammation, and consequently contribute to the disease progression and cap rupture risk. Nevertheless, many reciprocal relationships between macrophages and calcification remain to be exposed.

## Outlook

Throughout this review, we emphasized that the presence, characteristics, and iterative interplay of collagen, microcalcification, and macrophages have clear implications for tissue mechanics and consequently the risk of rupture. We believe that enhanced mechanistic insight into how biological processes change cap mechanical properties is a prerequisite for identifying the cap at risk of rupture.

**Fig. 3:**
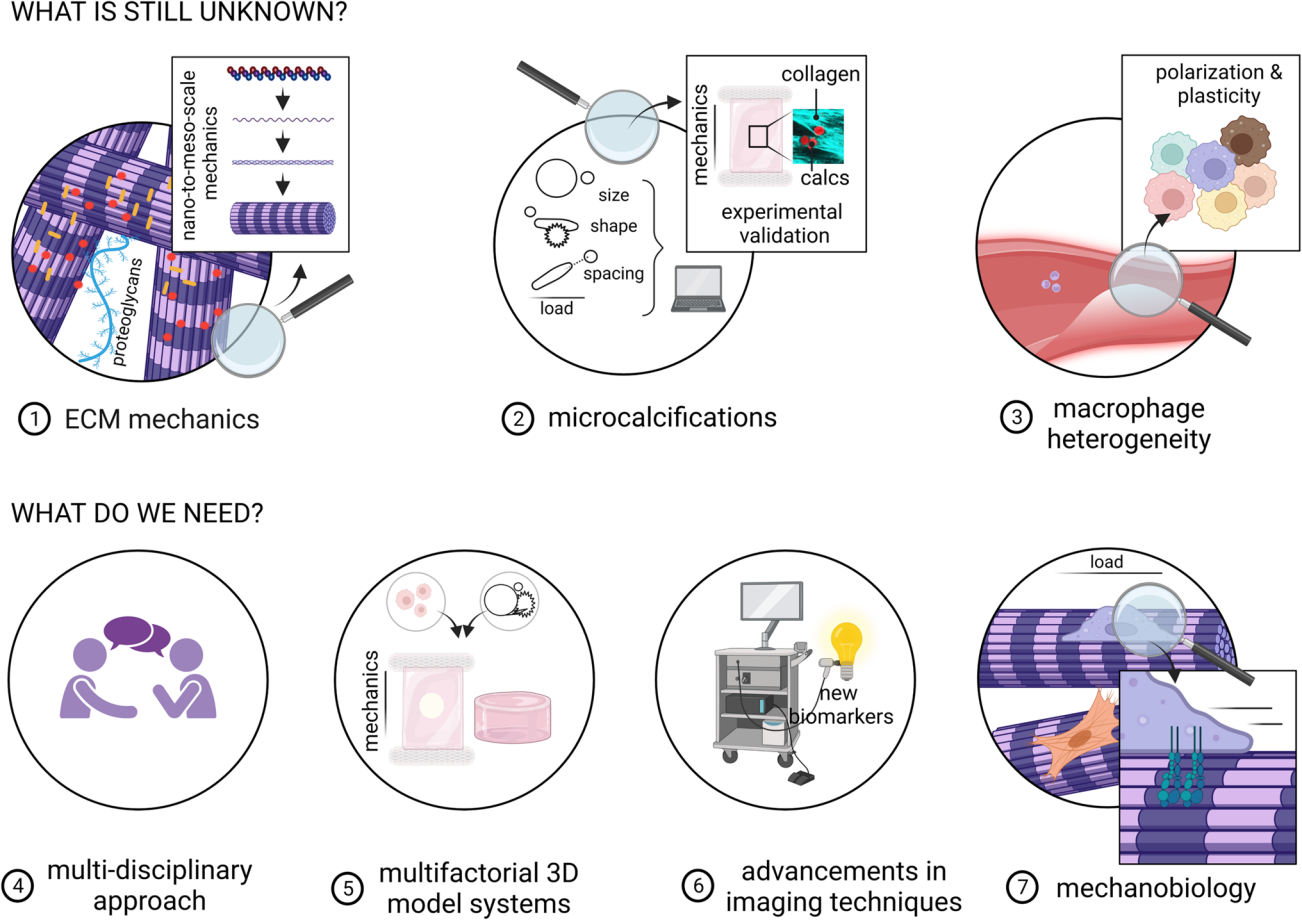
Overview of the knowledge that is currently still lacking and what is needed to improve cap rupture prediction. (1) For the understanding of ECM mechanics, collagen properties need to be assessed from nano-to-meso-scale as well as more in-depth analysis of other ECM components. (2) Microcalcifications need to be structurally investigated in experimental studies regarding size, shape, and spacing. (3) A better understanding of macrophage heterogeneity is needed. (4) A multidisciplinary approach is desired combining fields such as vascular biology and engineering. (5) Multifactorial 3D model systems are required to study more complex processes. (6) To be able to stratify findings to patients, advancements in imaging techniques are mandated. (7) Mechanobiology, the effect of cardiovascular mechanics on cellular behavior, should be the topic of research. Created with BioRender.com

In view of cap mechanics and the interplay between collagen, macrophages, and microcalcification, it can be concluded that much is still largely unknown (Fig. 3). Regarding collagen, emerging studies focus on the mechanical properties of collagen using a bottom-up approach studying nanoscale collagen mechanics [80], to be able to link this to mesoscale. In addition, increasing attention is being paid to the differences in global and local mechanical behavior [57, 241]. With respect to microcalcification, in-depth histological or micro-CT analyses of patient material, assessing microcalcification clustering, particle shape and density, are lacking. In addition, there is limited knowledge about microcalcification surface topography, which might greatly influence macrophage functionality [132] and consequently the collagenous matrix. Concerning macrophage heterogeneity, more athero-specific subsets are found to exist, mainly due to advances in single cell analysis [60], that provides a plethora of compounds affecting plaque properties and preventing rupture risk. The generally accepted statement that primarily pro-inflammatory macrophages are involved in rupture is obsolete as more atheroprotective subsets have been correlated with plaque vulnerability [19, 211]. A better distinction regarding macrophage phenotype and function should be made in relation to the environment to better understand macrophage-driven cap weakening. Furthermore, the impact of other ECM components (e.g., elastin, GAGs) [221, 230], or immune cells (non-macrophages) [61] needs to be assessed.

To fill the knowledge gaps, a holistic multidisciplinary approach is needed where the fields of immunopathology, vascular biology, biomechanics, tissue engineering, and biomedical engineering are combined. Many of the experimental studies discussed utilize animal, 2D in vitro or numerical models. Although informative and required, tissue content and mechanics are known to substantially diverge between animals and humans [97] and direct translation of 2D in vitro or numerical work to human cap mechanics is complex. To improve translatability and to validate computational approaches, more sophisticated 3D in vitro platforms are required that increasingly resemble pathophysiology, wherein human cells are exposed to various environmental factors simultaneously in a controlled manner (i.e., a multifactorial 3D in vitro model) [128]. Examples of such models include the collagen hydrogel that was utilized by Hutcheson et al. to mimic structural features of the atherosclerotic plaque to study microcalcification formation [106]. In addition, Mallone et al. bioengineered atherosclerotic plaques to investigate etiopathogenesis [158]. Our group recently made use of tissue engineering concepts to scrutinize relationships between tissue composition, the presence of microcalcifications, and mesoscale mechanical properties [109, 261]. Similar model systems can also be used to study patient variation [45, 121], as differences in age, sex, and co-morbidity are likely to affect tissue mechanics [91, 121]. Moreover, computational approaches combining models that describe blood flow and plaque deformation simultaneously could potentially provide new insights in the role of biomechanical factors in atherosclerosis [216].

To ultimately confirm findings and stratify them to patients, advanced imaging techniques are needed. Non-invasive imaging modalities including CT and MRI are currently the most widely used diagnostic tools in the clinic. However, invasive imaging techniques such as optical coherence tomography (OCT) and intravascular ultrasound (IVUS) can achieve higher resolution, and fusing these invasive techniques provides even further advancements for biomechanical modeling [90]. Recent hybrid imaging systems, such as PET–CT or PET–MRI can facilitate the detection of microcalcification and inflammation, to be able to create patient-specific modeling approaches and evaluate local mechanical properties [233]. Furthermore, specific tracers or diffusion tensor imaging can be used to visualize cap components, such as collagen type, maturity [192], and alignment [8, 239, 240].

Where this review elaborates on how cap compositional parameters affect mechanics, it is important to also obtain better mechanistic insight in how cell functionality and matrix properties are determined by the physical cues in their environment (i.e., the mechanobiology). It was shown that hemodynamic loading can determine macrophage phenotype [94] (add ref) and matrix content [27], among others by changing collagen’s susceptibility to degradation [77, 78]. It is, thus, likely that hemodynamic loading also influences other cap components such as microcalcifications, possibly altering their shape, size or topography.

Taken together, the creation of more complex, multifactorial 3D in vitro model systems as well as advancements in imaging techniques are required to better assess the impact of individual cap components as well as their reciprocal interplay on cap strength and vice versa. Obtained knowledge will ultimately serve as input to help in the diagnosis, prevention, treatment, and design of new therapies against atherosclerotic cap rupture.

## References

[1] Abd-Elrahman I, Meir K, Kosuge H, Ben-Nun Y, Sadan TW, Rubinstein C, Samet Y, McConnell MV, Blum G (2016). Characterizing cathepsin activity and macrophage subtypes in excised human carotid plaques. Stroke.

[2] Abdelbaky A, Corsini E, Figueroa AL, Fontanez S, Subramanian S, Ferencik M, Brady TJ, Hoffmann U, Tawakol A (2013). Focal arterial inflammation precedes subsequent calcification in the same location: a longitudinal FDG-PET/CT study. Circ Cardiovasc Imaging.

[3] Adachi E, Hayashi T (1986). In vitro formation of hybrid fibrils of type V collagen and type I collagen limited growth of type I collagen into thick fibrils by type V collagen. Connect Tissue Res.

[4] Adiguzel E, Ahmad PJ, Franco C, Bendeck MP (2009). Collagens in the progression and complications of atherosclerosis. Vasc Med.

[5] Aikawa E, Aikawa M, Rusanescu G, Iwamoto Y, Shi GP, Jaffer FA, Libby P, Figueiredo JL, Fukuda D, Kohler RH, Weissleder R (2009). Arterial and aortic valve calcification abolished by elastolytic cathepsin S deficiency in chronic renal disease. Circulation.

[6] Aikawa E, Nahrendorf M, Figueiredo JL, Swirski FK, Shtatland T, Kohler RH, Jaffer FA, Aikawa M, Weissleder R (2007). Osteogenesis associates with inflammation in early-stage atherosclerosis evaluated by molecular imaging in vivo. Circulation.

[7] Aimes RT, Quigley JP (1995). Matrix metalloproteinase-2 is an interstitial collagenase. Inhibitor-free enzyme catalyzes the cleavage of collagen fibrils and soluble native type I collagen generating the specific 3/4- and 1/4-length fragments. J Biol Chem.

[8] Akyildiz AC, Chai CK, Oomens CWJ, van der Lugt A, Baaijens FPT, Strijkers GJ, Gijsen FJH (2017). 3D fiber orientation in atherosclerotic carotid plaques. J Struct Biol.

[9] Akyildiz AC, Speelman L, Gijsen FJH (2014). Mechanical properties of human atherosclerotic intima tissue. J Biomech.

[10] Amento EP, Ehsani N, Palmer H, Libby P (1991). Cytokines and growth factors positively and negatively regulate interstitial collagen gene expression in human vascular smooth muscle cells. Arterioscler Thromb Vasc Biol.

[11] Arroyo LH, Lee RT (1999). Mechanisms of plaque rupture: mechanical and biologic interactions. Cardiovasc Res.

[12] Asgari M, Latifi N, Heris HK, Vali H, Mongeau L (2017). In vitro fibrillogenesis of tropocollagen type III in collagen type I affects its relative fibrillar topology and mechanics. Sci Rep.

[13] Balguid A, Rubbens MP, Mol A, Bank RA, Bogers AJJC, Van Kats JP, De Mol BAJM, Baaijens FPT, Bouten CVC (2007). The role of collagen cross-links in biomechanical behavior of human aortic heart valve leaflets—relevance for tissue engineering. Tissue Eng.

[14] Barnes MJ, Farndale RW (1999). Collagens and atherosclerosis. Exp Gerontol.

[15] Barrett HE, Van der Heiden K, Farrell E, Gijsen FJH, Akyildiz AC (2019). Calcifications in atherosclerotic plaques and impact on plaque biomechanics. J Biomech.

[16] Bartlett B, Ludewick HP, Misra A, Lee S, Dwivedi G (2019). Macrophages and t cells in atherosclerosis: a translational perspective. Am J Physiol- Hear Circ Physiol.

[17] Basatemur GL, Jørgensen HF, Clarke MCH, Bennett MR, Mallat Z (2019). Vascular smooth muscle cells in atherosclerosis. Nat Rev Cardiol.

[18] Bauriedel G, Hutter R, Welsch U, Bach R, Sievert H, Lüderitz B (1999). Role of smooth muscle cell death in advanced coronary primary lesions: implications for plaque instability. Cardiovasc Res.

[19] Bengtsson E, Hultman K, Edsfeldt A, Persson A, Nitulescu M, Nilsson J, Gonçalves I, Björkbacka H (2020). CD163+ macrophages are associated with a vulnerable plaque phenotype in human carotid plaques. Sci Rep.

[20] Bentzon JF, Otsuka F, Virmani R, Falk E (2014). Mechanisms of plaque formation and rupture. Circ Res.

[21] Bidanset DJ, Guidry C, Rosenberg LC, Choi HU, Timpl R, Hook M (1992). Binding of the proteoglycan decorin to collagen type VI. J Biol Chem.

[22] Bigg HF, Rowan AD, Barker MD, Cawston TE (2007). Activity of matrix metalloproteinase-9 against native collagen types I and III. FEBS J.

[23] Birk DE, Silver FH (1984). Collagen fibrillogenesis in vitro: comparison of types I, II, and III. Arch Biochem Biophys.

[24] Biswas SK, Mantovani A (2010). Macrophage plasticity and interaction with lymphocyte subsets: cancer as a paradigm. Nat Immunol.

[25] Bluestein D, Alemu Y, Avrahami I, Gharib M, Dumont K, Ricotta JJ, Einav S (2008). Influence of microcalcifications on vulnerable plaque mechanics using FSI modeling. J Biomech.

[26] Bobryshev YV, Ivanova EA, Chistiakov DA, Nikiforov NG, Orekhov AN (2016). Macrophages and their role in atherosclerosis: pathophysiology and transcriptome analysis. Biomed Res Int.

[27] Boerboom R, Rubbens MP, Driessen NJB, Bouten CVC, Baaijens FPT (2008). Effect of strain magnitude on the tissue properties of engineered cardiovascular constructs. Ann Biomed Eng.

[28] Borrelli V, di Marzo L, Sapienza P, Colasanti M, Moroni E, Cavallaro A (2006). Role of platelet-derived growth factor and transforming growth factor β1 the in the regulation of metalloproteinase expressions. Surgery.

[29] Borza CM, Pozzi A (2014). Discoidin domain receptors in disease. Matrix Biol.

[30] Boshuizen MCS, De Winther MPJ (2015). Interferons as essential modulators of atherosclerosis. Arterioscler Thromb Vasc Biol.

[31] Bourne LE, Wheeler-Jones CPD, Orriss IR (2021). Regulation of mineralisation in bone and vascular tissue: a comparative review. J Endocrinol.

[32] Boyle JJ, Harrington HA, Piper E, Elderfield K, Stark J, Landis RC, Haskard DO (2009). Coronary intraplaque hemorrhage evokes a novel atheroprotective macrophage phenotype. Am J Pathol.

[33] Boyle JJ, Weissberg PL, Bennett MR (2003). Tumor necrosis factor-alpha promotes macrophage-induced vascular smooth muscle cell apoptosis by direct and autocrine mechanisms. Arterioscler Thromb Vasc Biol.

[34] Buckley MR, Evans E, Satchel LN, Matuszewski PE, Chen Y-L, Elliott DM, Soslowsky LJ, Dodge GR (2013). Distributions of types I, II and III collagen by region in the human supraspinatus tendon. Connect Tissue Res.

[35] Burgmaier M, Milzi A, Dettori R, Burgmaier K, Marx N, Reith S (2018). Co-localization of plaque macrophages with calcification is associated with a more vulnerable plaque phenotype and a greater calcification burden in coronary target segments as determined by OCT. PLoS ONE.

[36] Burke AP, Farb A, Malcom GT, Liang YH, Smialek JE, Virmani R (1999). Plaque rupture and sudden death related to exertion in men with coronary artery disease. J Am Med Assoc.

[37] Burleigh MC, Brigfs AD, Lendon CL, Davies MJ, Born GVR, Richardson PD (1992). Collagen types I and III, collagen content, GAGs and mechanical strength of human atherosclerotic plaque caps: span-wise variations. Atherosclerosis.

[38] Byon CH, Sun Y, Chen J, Yuan K, Mao X, Heath JM, Anderson PG, Tintut Y, Demer LL, Wang D, Chen Y (2011). Runx2-upregulated receptor activator of nuclear factor κB ligand in calcifying smooth muscle cells promotes migration and osteoclastic differentiation of macrophages. Arterioscler Thromb Vasc Biol.

[39] Cameron JN, Mehta OH, Michail M, Chan J, Nicholls SJ, Bennett MR, Brown AJ (2020). Exploring the relationship between biomechanical stresses and coronary atherosclerosis. Atherosclerosis.

[40] Canet-Soulas E, Bessueille L, Mechtouff L, Magne D (2021). The elusive origin of atherosclerotic plaque Calcification. Front Cell Dev Biol.

[41] Cardoso L, Kelly-Arnold A, Maldonado N, Laudier D, Weinbaum S (2014). Effect of tissue properties, shape and orientation of microcalcifications on vulnerable cap stability using different hyperelastic constitutive models. J Biomech.

[42] Cardoso L, Weinbaum S (2021). Microcalcifications and plaque rupture. Biomech Coron Atheroscler Plaque From Model Patient.

[43] Chai CK, Akyildiz AC, Speelman L, Gijsen FJH, Oomens CWJ, van Sambeek MRHM, van der Lugt A, Baaijens FPT (2013). Local axial compressive mechanical properties of human carotid atherosclerotic plaques-characterisation by indentation test and inverse finite element analysis. J Biomech.

[44] Chatrou MLL, Cleutjens JP, Van Vusse GJD, Roijers RB, Mutsaers PHA, Schurgers LJ (2015). Intra-section analysis of human coronary arteries reveals a potential role for micro-calcifications in macrophage recruitment in the early stage of atherosclerosis. PLoS One.

[45] Chen H, Kassab GS (2016). Microstructure-based biomechanics of coronary arteries in health and disease. J Biomech.

[46] Chen L, Qiao P, Liu H, Shao L (2020). Amorphous calcium phosphate NPs mediate the macrophage response and modulate BMSC osteogenesis. Inflamm.

[47] Chen NX, O’Neill KD, Chen X, Moe SM (2008). Annexin-mediated matrix vesicle calcification in vascular smooth muscle cells. J Bone Miner Res.

[48] Chen Y, Waqar AB, Nishijima K, Ning B, Kitajima S, Matsuhisa F, Chen L, Liu E, Koike T, Yu Y, Zhang J, Chen YE, Sun H, Liang J, Fan J (2020). Macrophage-derived MMP-9 enhances the progression of atherosclerotic lesions and vascular calcification in transgenic rabbits. J Cell Mol Med.

[49] Chiu JJ, Usami S, Chien S (2008). Vascular endothelial responses to disturbed flow: Pathologic implications for atherosclerosis. Bioeng Cell Tissue Res.

[50] Cho HJ, Cho HJ, Kim HS (2009). Osteopontin: a multifunctional protein at the crossroads of inflammation, atherosclerosis, and vascular calcification. Curr Atheroscler Reports.

[51] Chu B, Kampschulte A, Ferguson MS, Kerwin WS, Yarnykh VL, O’Brien KD, Polissar NL, Hatsukami TS, Yuan C (2004). Hemorrhage in the atherosclerotic carotid plaque: a high-resolution MRI study. Stroke.

[52] Cilla M, Monterde D, Pena E, Martinez MA (2013). Does microcalcification increase the risk of rupture?. Proc Inst Mech Eng Part H J Eng Med.

[53] Clarke MCH, Littlewood TD, Figg N, Maguire JJ, Davenport AP, Goddard M, Bennett MR (2008). Chronic apoptosis of vascular smooth muscle cells accelerates atherosclerosis and promotes calcification and medial degeneration. Circ Res.

[54] Colin S, Chinetti-Gbaguidi G (2014). Staels B (2014) Macrophage phenotypes in atherosclerosis. Immunol Rev.

[55] Corti A, Khalil D, De Paolis A, Cardoso L (2023). Size and proximity of micro-scale hard-inclusions increase the risk of rupture in fibroatheroma-like laboratory models. J Mech Behav Biomed Mater.

[56] Corti A, De Paolis A, Grossman P, Dinh PA, Aikawa E, Weinbaum S, Cardoso L (2022). The effect of plaque morphology, material composition and microcalcifications on the risk of cap rupture: a structural analysis of vulnerable atherosclerotic plaques. Front Cardiovasc Med.

[57] Crielaard H, Torun SG, Wissing TB, Muñoz PM, Kremers GJ, Gijsen FJH, Van Der Heiden K, Akyildiz AC (2022). A method to study the correlation between local collagen structure and mechanical properties of atherosclerotic plaque fibrous tissue. J Vis Exp.

[58] Daghem M, Bing R, Fayad ZA, Dweck MR (2020). Noninvasive imaging to assess atherosclerotic plaque composition and disease activity: coronary and carotid applications. JACC Cardiovasc Imaging.

[59] Davis S, Carsten S, Sutton L (2016). Characterization of fracture behavior of human atherosclerotic fibrous caps using a miniature single edge notched tensile test. Acta Biomater.

[60] Decano JL, Aikawa M (2018). Dynamic macrophages: understanding mechanisms of activation as guide to therapy for atherosclerotic vascular disease. Front Cardiovasc Med.

[61] Depuydt MAC, Prange KHM, Slenders L, Örd T, Elbersen D, Boltjes A, De Jager SCA, Asselbergs FW, De Borst GJ, Aavik E, Lönnberg T, Lutgens E, Glass CK, Den Ruijter HM, Kaikkonen MU, Bot I, Slütter B, Van Der Laan SW, Yla-Herttuala S, Mokry M, Kuiper J, De Winther MPJ, Pasterkamp G (2020). Microanatomy of the human atherosclerotic plaque by single-cell transcriptomics. Circ Res.

[62] Dhawan SS, Avati Nanjundappa RP, Branch JR, Taylor WR, Quyyumi AA, Jo H, Mcdaniel MC, Suo J, Coulter WH, Giddens D, Samady H (2010). Shear stress and plaque development. Expert Rev Cardiovasc Ther.

[63] Dollery CM, Owen CA, Sukhova GK, Krettek A, Shapiro SD, Libby P (2003). Neutrophil elastase in human atherosclerotic plaques production by macrophages. Circulation.

[64] Douglas GR, Brown AJ, Gillard JH, Bennett MR, Sutcliffe MPF, Teng Z (2017). Impact of fiber structure on the material stability and rupture mechanisms of coronary atherosclerotic plaques. Ann Biomed Eng.

[65] Durham AL, Speer MY, Scatena M, Giachelli CM, Shanahan CM (2018). Role of smooth muscle cells in vascular calcification: implications in atherosclerosis and arterial stiffness. Cardiovasc Res.

[66] Dweck MR, Maurovich-Horvat P, Leiner T, Cosyns B, Fayad ZA, Gijsen FJH, Van Der Heiden K, Kooi ME, Maehara A, Muller JE, Newby DE, Narula J, Pontone G, Regar E, Serruys PW, Van Der Steen AFW, Stone PH, Waltenberger JL, Yuan C, Evans PC, Lutgens E, Wentzel JJ, Bäck M (2020). Contemporary rationale for non-invasive imaging of adverse coronary plaque features to identify the vulnerable patient: a position paper from the European Society of Cardiology Working Group on Atherosclerosis and Vascular Biology and the European Associa. Eur Heart J Cardiovasc Imaging.

[67] Ewence AE, Bootman M, Roderick HL, Skepper JN, McCarthy G, Epple M, Neumann M, Shanahan CM, Proudfoot D (2008). Calcium phosphate crystals induce cell death in human vascular smooth muscle cells. Circ Res.

[68] Ferencik M, Mayrhofer T, Bittner DO, Emami H, Puchner SB, Lu MT, Meyersohn NM, Ivanov AV, Adami EC, Patel MR, Mark DB, Udelson JE, Lee KL, Douglas PS, Hoffmann U (2018). Use of high-risk coronary atherosclerotic plaque detection for risk stratification of patients with stable chest pain. JAMA Cardiol.

[69] Fessel G, Li Y, Diederich V, Guizar-Sicairos M, Schneider P, Sell DR, Monnier VM, Snedeker JG (2014). Advanced glycation end-products reduce collagen molecular sliding to affect collagen fibril damage mechanisms but not stiffness. PLoS ONE.

[70] Fields GB (1991). A model for interstitial collagen catabolism by mammalian collagenases. J theor Biol.

[71] Fields GB (2013). Interstitial collagen catabolism. J Biol Chem.

[72] Finn AV, Nakano M, Narula J, Kolodgie FD, Virmani R (2010). Concept of vulnerable/unstable plaque. Arterioscler Thromb Vasc Biol.

[73] Fonović M, Turk B (2014). Cysteine cathepsins and extracellular matrix degradation. Biochim Biophys Acta - Gen Subj.

[74] Franco CD, Hou G, Bendeck MP (2002). Collagens, integrins, and the discoidin domain receptors in arterial occlusive disease. Trends Cardiovasc Med.

[75] Fu Y, Gao C, Liang Y, Wang M, Huang Y, Ma W, Li T, Jia Y, Yu F, Zhu W, Cui Q, Li Y, Xu Q, Wang X, Kong W (2016). Shift of macrophage phenotype due to cartilage oligomeric matrix protein deficiency drives atherosclerotic calcification. Circ Res.

[76] de Gaetano M, Crean D, Barry M, Belton O (2016). M1- and M2-type macrophage responses are predictive of adverse outcomes in human atherosclerosis. Front Immunol.

[77] Gaul RT, Nolan DR, Lally C (2018). The use of small angle light scattering in assessing strain induced collagen degradation in arterial tissue ex vivo. J Biomech.

[78] Gaul RT, Nolan DR, Ristori T, Bouten CVC, Loerakker S, Lally C (2018). Strain mediated enzymatic degradation of arterial tissue: insights into the role of the non-collagenous tissue matrix and collagen crimp. Acta Biomater.

[79] Gautieri A, Passini FS, Silván U, Guizar-Sicairos M, Carimati G, Volpi P, Moretti M, Schoenhuber H, Redaelli A, Berli M, Snedeker JG (2017). Advanced glycation end-products: mechanics of aged collagen from molecule to tissue. Matrix Biol.

[80] Gautieri A, Vesentini S, Redaelli A, Buehler MJ (2011). Hierarchical structure and nanomechanics of collagen microfibrils from the atomistic scale up. Nano Lett.

[81] Geng YJ, Libby P (1995). Evidence for apoptosis in advanced human atheroma: colocalization with interleukin-1β-converting enzyme. Am J Pathol.

[82] Gialeli C, Shami A, Gonçalves I (2021). Extracellular matrix: paving the way to the newest trends in atherosclerosis. Curr Opin Lipidol.

[83] Gijsen FJH, Vis B, Barrett HE, Zadpoor AA, Verhagen HJ, Bos D, Van Der Steen AFW, Akyildiz AC (2021). Morphometric and mechanical analyses of calcifications and fibrous plaque tissue in carotid arteries for plaque rupture risk assessment. IEEE Trans Biomed Eng.

[84] Gijsen FJH, Wentzel JJ, Thury A, Mastik F, Schaar JA, Schuurbiers JCH, Slager CJ, Van Der Giessen WJ, De Feyter PJ, Van Der Steen AFW, Serruys PW (2008). Strain distribution over plaques in human coronary arteries relates to shear stress. Am J Physiol.

[85] Goel S, Miller A, Agarwal C, Zakin E, Acholonu M, Gidwani U, Sharma A, Kulbak G, Shani J, Chen O (2015). Imaging modalities to identity inflammation in an atherosclerotic plaque. Radiol Res Pract.

[86] Van Goethem E, Poincloux R, Gauffre F, Maridonneau-Parini I, Le Cabec V (2010). Matrix architecture dictates three-dimensional migration modes of human macrophages: differential involvement of proteases and podosome-like structures. J Immunol.

[87] Goldin A, Beckman JA, Schmidt AM, Creager MA (2006). Advanced glycation end products: sparking the development of diabetic vascular injury. Circulation.

[88] Gourgas O, Marulanda J, Zhang P, Murshed M, Cerruti M (2018). Multidisciplinary approach to understand medial arterial calcification. Arterioscler Thromb Vasc Biol.

[89] Greaves DR, Gordon S (2001). Immunity, atherosclerosis and cardiovascular disease. Trends Immunol.

[90] Guo X, Giddens DP, Molony D, Yang C, Samady H, Zheng J, Mintz G, Maehara A, Wang L, Pei X, Li Z-Y, Tang D (2017). An FSI modeling approach to combine IVUS and OCT for more accurate patient-specific coronary cap thickness and stress/strain calculations. J Biomech Eng.

[91] Hansen F, Mangell P, Sonesson B, Länne T (1995). Diameter and compliance in the human common carotid artery—variations with age and sex. Ultrasound Med Biol.

[92] Hanssen NMJ, Wouters K, Huijberts MS, Gijbels MJ, Sluimer JC, Scheijen JLJM, Heeneman S, Biessen EAL, Daemen MJAP, Brownlee M, De Kleijn DP, Stehouwer CDA, Pasterkamp G, Schalkwijk CG (2014). Higher levels of advanced glycation endproducts in human carotid atherosclerotic plaques are associated with a rupture-prone phenotype. Eur Heart J.

[93] Hansson GK, Hellstrand M, Rymo L, Rubbia L, Gabbiani G (1989). Interferon γ inhibits both proliferation and expression of differentiation-specific α-smooth muscle actin in arterial smooth muscle cells. J Exp Med.

[94] Haschak M, LoPresti S, Stahl E, Dash S, Popovich B, Brown BN (2021). Macrophage phenotype and function are dependent upon the composition and biomechanics of the local cardiac tissue microenvironment. Aging (Albany NY).

[95] He L, Zhang CL, Chen Q, Wang L, Huang Y (2022). Endothelial shear stress signal transduction and atherogenesis: from mechanisms to therapeutics. Pharmacol Ther.

[96] Heeneman S, Cleutjens JP, Faber BC, Creemers EE, van Suylen RJ, Lutgens E, Cleutjens KB, Daemen MJ (2003). The dynamic extracellular matrix: intervention strategies during heart failure and atherosclerosis. J Pathol.

[97] Van der Heiden K, Hoogendoorn A, Daemen MJ, Gijsen FJH (2016). Animal models for plaque rupture: a biomechanical assessment. Thromb Haemost.

[98] Herisson F, Heymann MF, Chétiveaux M, Charrier C, Battaglia S, Pilet P, Rouillon T, Krempf M, Lemarchand P, Heymann D, Gouëffic Y (2011). Carotid and femoral atherosclerotic plaques show different morphology. Atherosclerosis.

[99] Herrington W, Lacey B, Sherliker P, Armitage J, Lewington S (2016). Epidemiology of atherosclerosis and the potential to reduce the global burden of atherothrombotic disease. Circ Res.

[100] Holzapfel GA, Gasser TC, Stadler M (2002). A structural model for the viscoelastic behavior of arterial walls: continuum formulation and finite element analysis. Eur J Mech A/Solids.

[101] Holzapfel GA, Mulvihill JJ, Cunnane EM, Walsh MT (2014). Computational approaches for analyzing the mechanics of atherosclerotic plaques: a review. J Biomech.

[102] Hoop CL, Zhu J, Nunes AM, Case DA, Baum J (2017). Revealing accessibility of cryptic protein binding sites within the functional collagen fibril. Biomolecules.

[103] Humphrey JD (2002). Cardiovascular solid mechanics: cells, tissues, and organs.

[104] Husain T, Abbott CR, Scott DJA, Gough MJ (1999). Macrophage accumulation within the cap of carotid atherosclerotic plaques is associated with the onset of cerebral ischemic events. J Vasc Surg.

[105] Hutcheson JD, Goettsch C, Bertazzo S, Maldonado N, Ruiz JL, Goh W, Yabusaki K, Faits T, Bouten C, Franck G, Quillard T, Libby P, Aikawa M, Weinbaum S, Aikawa E (2016). Genesis and growth of extracellular-vesicle-derived microcalcification in atherosclerotic plaques. Nat Mater.

[106] Hutcheson JD, Maldonado N, Aikawa E (2014). Small entities with large impact: microcalcifications and atherosclerotic plaque vulnerability. Curr Opin Lipidol.

[107] Ikeda K, Souma Y, Akakabe Y, Kitamura Y, Matsuo K, Shimoda Y, Ueyama T, Matoba S, Yamada H, Okigaki M, Matsubara H (2012). Macrophages play a unique role in the plaque calcification by enhancing the osteogenic signals exerted by vascular smooth muscle cells. Biochem Biophys Res Commun.

[108] Jang IK, Tearney GJ, MacNeill B, Takano M, Moselewski F, Iftima N, Shishkov M, Houser S, Aretz HT, Halpern EF, Bouma BE (2005). In vivo characterization of coronary atherosclerotic plaque by use of optical coherence tomography. Circulation.

[109] Jansen I, Crielaard H, Wissing T, Bouten C, Gijsen F, Farrell E, van der Heiden K (2023). A tissue-engineered model of the atherosclerotic plaque cap: towards understanding the role of micro-calcifications in plaque rupture. APL Bioeng. DOI.

[110] Jinnouchi H, Guo L, Sakamoto A, Torii S, Sato Y, Cornelissen A, Kuntz S, Paek KH, Fernandez R, Fuller D, Gadhoke N, Surve D, Romero M, Kolodgie FD, Virmani R, Finn AV (2020). Diversity of macrophage phenotypes and responses in atherosclerosis. Cell Mol Life Sci.

[111] Johnston RD, Gaul RT, Lally C (2021). An investigation into the critical role of fibre orientation in the ultimate tensile strength and stiffness of human carotid plaque caps. Acta Biomater.

[112] Jover E, Silvente A, Marín F, Martínez-González J, Orriols M, Martinez CM, Puche CM, Valdés M, Rodriguez C, Hernández-Romero D (2018). Inhibition of enzymes involved in collagen cross-linking reduces vascular smooth muscle cell calcification. FASEB J.

[113] Kapustin AN, Davies JD, Reynolds JL, McNair R, Jones GT, Sidibe A, Schurgers LJ, Skepper JN, Proudfoot D, Mayr M, Shanahan CM (2011). Calcium regulates key components of vascular smooth muscle cell-derived matrix vesicles to enhance mineralization. Circ Res.

[114] Katsuda S, Okada Y, Minamoto T, Oda Y, Matsui Y, Nakanishi I (1992). Collagens in human atherosclerosis: immunohistochemical analysis using collagen type-specific antibodies. Arterioscler Thromb.

[115] Kawakami R, Katsuki S, Travers R, Romero DC, Becker-Greene D, Passos LSA, Higashi H, Blaser MC, Sukhova GK, Buttigieg J, Kopriva D, Schmidt AM, Anderson DG, Singh SA, Cardoso L, Weinbaum S, Libby P, Aikawa M, Croce K, Aikawa E (2020). S100A9-RAGE axis accelerates formation of macrophage-mediated extracellular vesicle microcalcification in diabetes. Arterioscler Thromb Vasc Biol.

[116] Kelly-Arnold A, Maldonado N, Laudier D, Aikawa E, Cardoso L, Weinbaum S (2013). Revised microcalcification hypothesis for fibrous cap rupture in human coronary arteries. Proc Natl Acad Sci USA.

[117] Kishore U, Italiani P, Joshua M, Braga TT, Sebastian J, Agudelo H, Olsen N, Camara S (2015). Macrophages during the fibrotic process: M2 as friend and foe. Front Immunol.

[118] Koga J, Aikawa M (2012). Crosstalk between macrophages and smooth muscle cells in atherosclerotic vascular diseases. Vascul Pharmacol.

[119] Kolodgie FD, Burke AP, Farb A, Gold HK, Yuan J, Narula J, Finn AV, Virmani R (2001). The thin-cap fibroatheroma: a type of vulnerable plaque the major precursor lesion to acute coronary syndromes. Curr Opin Cardiol.

[120] Kolodgie FD, Narula J, Burke AP, Haider N, Farb A, Hui-Liang Y, Smialek J, Virmani R (2000). Localization of apoptotic macrophages at the site of plaque rupture in sudden coronary death. Am J Pathol.

[121] de Kort BJ, Koch SE, Wissing TB, Krebber MM, Bouten CVC, Smits AIPM (2021). Immuno-regenerative biomaterials for in situ cardiovascular tissue engineering—Do patient characteristics warrant precision engineering?. Adv Drug Deliv Rev.

[122] De Korte CL, Sierevogel MJ, Mastik F, Strijder C, Schaar JA, Velema E, Pasterkamp G, Serruys PW, Van der Steen AFW (2002). Identification of atherosclerotic plaque components with intravascular ultrasound elastography in vivo: a Yucatan pig study. Circulation.

[123] Krettek A, Sukhova GK, Libby P (2003). Elastogenesis in human arterial disease: a role for macrophages in disordered elastin synthesis. Arterioscler Thromb Vasc Biol.

[124] Krohn JB, Hutcheson JD, Martínez-Martínez E, Aikawa E (2016). Extracellular vesicles in cardiovascular calcification: expanding current paradigms. J Physiol.

[125] Krohn JB, Hutcheson JD, Martínez-Martínez E, Irvin WS, Bouten CVC, Bertazzo S, Bendeck MP, Aikawa E (2016). Discoidin domain receptor-1 regulates calcific extracellular vesicle release in vascular smooth muscle cell fibrocalcific response via transforming growth factor-β signaling. Arterioscler Thromb Vasc Biol.

[126] Krotova K, Khodayari N, Oshins R, Aslanidi G, Brantly ML (2020). Neutrophil elastase promotes macrophage cell adhesion and cytokine production through the integrin-Src kinases pathway. Sci Rep.

[127] Kumar A, Kar S, Fay WP (2011). Thrombosis, physical activity, and acute coronary syndromes. J Appl Physiol.

[128] Kurniawan NA, Bouten CVC (2018). Mechanobiology of the cell–matrix interplay: catching a glimpse of complexity via minimalistic models. Extrem Mech Lett.

[129] Kusmartsev S, Dominguez-Gutierrez PR, Canales BK, Bird VG, Vieweg J, Khan SR (2016). Calcium oxalate stone fragment and crystal phagocytosis by human macrophages. J Urol.

[130] Laquerriere P, Grandjean-Laquerriere A, Jallot E, Balossier G, Frayssinet P, Guenounou M (2003). Importance of hydroxyapatite particles characteristics on cytokines production by human monocytes in vitro. Biomaterials.

[131] Larsen AMH, Kuczek DE, Kalvisa A, Siersbæk MS, Thorseth M-L, Johansen AZ, Carretta M, Grøntved L, Vang O, Madsen DH (2020). Collagen density modulates the immunosuppressive functions of macrophages. J Immunol.

[132] Lebre F, Sridharan R, Sawkins MJ, Kelly DJ, O’Brien FJ, Lavelle EC (2017). The shape and size of hydroxyapatite particles dictate inflammatory responses following implantation. Sci Reports.

[133] Lee JS, Morrisett JD, Tung C-H, Tung CH (2012). Detection of hydroxyapatite in calcified cardiovascular tissues. Atherosclerosis.

[134] Lee SJ, Lee IK, Jeon JH (2020). Vascular calcification—new insights into its mechanism. Int J Mol Sci.

[135] Lendon CL, Briggs AD, Born GVR, Burleigh MC, Davies MJ (1988). Mechanical testing of connective tissue in the search for determinants of atherosclerotic plaque cap rupture. Biochem Soc Trans.

[136] Lendon CL, Davies MJ, Born GVR, Richardson PD (1991). Atherosclerotic plaque caps are locally weakened when macrophages density is increased. Atherosclerosis.

[137] Leopold JA (2015). Vascular calcification: mechanisms of vascular smooth muscle cell calcification. Trends Cardiovasc Med.

[138] Lepidi S, Kenagy RD, Raines EW, Chiu ES, Chait A, Ross R, Clowes AW (2001). MMP9 production by human monocyte-derived macrophages is decreased on polymerized type I collagen. J Vasc Surg.

[139] Leszczynska A, O’Doherty A, Farrell E, Pindjakova J, O’Brien FJ, O’Brien T, Barry F, Murphy M (2016). Differentiation of vascular stem cells contributes to ectopic calcification of atherosclerotic plaque. Stem Cells.

[140] Li P, Wang Y, Liu X, Liu B, Wang Z, Xie F, Qiao W, Liang E, Lu Q, Zhang M (2020). Loss of PARP-1 attenuates diabetic arteriosclerotic calcification via Stat1/Runx2 axis. Cell Death Dis.

[141] Li R, Mittelstein D, Lee J, Fang K, Majumdar R, Tintut Y, Demer LL, Hsiai TK (2012). A dynamic model of calcific nodule destabilization in response to monocyteand oxidized lipid-induced matrix metalloproteinases. Am J Physiol.

[142] Li Z, Yasuda Y, Li W, Bogyo M, Katz N, Gordon RE, Fields GB, Brömme D (2004). Regulation of collagenase activities of human cathepsins by glycosaminoglycans. J Biol Chem.

[143] Libby P (2012). Inflammation in atherosclerosis. Arterioscler Thromb Vasc Biol.

[144] Libby P, Buring JE, Badimon L, Hansson GK, Deanfield J, Bittencourt MS, Tokgözoğlu L, Lewis EF (2019). Atherosclerosis. Nat Rev Dis Prim.

[145] Libby P, Geng Y, Aikawa M, Schoenbeck U, Mach F, Clinton SK, Sukhova GK, Lee RT (1996). Macrophages and atherosclerotic plaque stability. Curr Opin Lipidol.

[146] Libby P, Lichtman AH, Hansson GK (2013). Immune effector mechanisms implicated in atherosclerosis: from mice to humans. Immunity.

[147] Libby P, Ridker PM, Maseri A (2002). Inflammation and atherosclerosis. Circulation.

[148] Lin ME, Chen TM, Wallingford MC, Nguyen NB, Yamada S, Sawangmake C, Zhang J, Speer MY, Giachelli CM (2016). Runx2 deletion in smooth muscle cells inhibits vascular osteochondrogenesis and calcification but not atherosclerotic lesion formation. Cardiovasc Res.

[149] Lin P, Ji HH, Li YJ, Guo SD (2021). Macrophage plasticity and atherosclerosis therapy. Front Mol Biosci.

[150] Lin TC, Tintut Y, Lyman A, MacK W, Demer LL, Hsiai TK (2006). Mechanical response of a calcified plaque model to fluid shear force. Ann Biomed Eng.

[151] Lucero HA, Kagan HM (2006). Lysyl oxidase: an oxidative enzyme and effector of cell function. Cell Mol Life Sci.

[152] Lutgens E, Van Suylen RJ, Faber BC, Gijbels MJ, Eurlings PM, Bijnens AP, Cleutjens KB, Heeneman S, Daemen MJAP (2003). Atherosclerotic plaque rupture: local or systemic process?. Arterioscler Thromb Vasc Biol.

[153] Maehara A, Mintz GS, Bui AB, Walter OR, Castagna MT, Canos D, Pichard AD, Satler LF, Waksman R, Suddath WO, Laird JR, Kent KM, Weissman NJ (2002). Morphologic and angiographic features of coronary plaque rupture detected by intravascular ultrasound. J Am Coll Cardiol.

[154] Mäki JM, Sormunen R, Lippo S, Kaarteenaho-Wiik R, Soininen R, Myllyharju J (2005). Lysyl oxidase is essential for normal development and function of the respiratory system and for the integrity of elastic and collagen fibers in various tissues. Am J Pathol.

[155] Maldonado K-A, Cardosa W (2013). The explosive growth of small voids in vulnerable cap rupture; cavitation and interfacial debonding. J Biomech.

[156] Maldonado N, Kelly-Arnold A, Laudier D, Weinbaum S, Cardoso L (2015). Imaging and analysis of microcalcifications and lipid/necrotic core calcification in fibrous cap atheroma. Int J Cardiovasc Imaging.

[157] Maldonado N, Kelly-Arnold A, Vengrenyuk Y, Laudier D, Fallon JT, Virmani R, Cardoso L, Weinbaum S (2012). A mechanistic analysis of the role of microcalcifications in atherosclerotic plaque stability: potential implications for plaque rupture. Am J Physiol.

[158] Mallone A, Stenger C, Von Eckardstein A, Hoerstrup SP, Weber B (2018). Biofabricating atherosclerotic plaques: in vitro engineering of a three-dimensional human fibroatheroma model. Biomaterials.

[159] Martínez-González J, Varona S, Cañes L, Galán M, Briones AM, Cachofeiro V, Rodríguez C (2019). Emerging roles of lysyl oxidases in the cardiovascular system: new concepts and therapeutic challenges. Biomolecules.

[160] McElroy M, Kim Y, Niccoli G, Vergallo R, Langford-Smith A, Crea F, Gijsen F, Johnson T, Keshmiri A, White SJ (2021). Identification of the haemodynamic environment permissive for plaque erosion. Sci Rep.

[161] Molnar J, Fong KSK, He QP, Hayashi K, Kim Y, Fong SFT, Fogelgren B, Szauter KM, Mink M, Csiszar K (2003). Structural and functional diversity of lysyl oxidase and the LOX-like proteins. Biochim Biophys Acta.

[162] Montanaro M, Scimeca M, Anemona L, Servadei F, Giacobbi E, Bonfiglio R, Bonanno E, Urbano N, Ippoliti A, Santeusanio G, Schillaci O, Mauriello A (2021). The paradox effect of calcification in carotid atherosclerosis: microcalcification is correlated with plaque instability. Int J Mol Sci.

[163] Moreno F, Palacios N, Fuster F (1994). Macrophage infiltration in acute coronary syndromes: implications for plaque rupture. Circulation.

[164] Mori H, Torii S, Kutyna M, Sakamoto A, Finn AV, Virmani R (2018). Coronary artery calcification and its progression: What does it really mean?. JACC Cardiovasc Imaging.

[165] Morton LF, Barnes MJ (1982). Collagen polymorphism in the normal and diseased blood vessel wall investigation of collagens types I, III and V. Atherosclerosis.

[166] Moss AJ, Sim AM, Adamson PD, Seidman MA, Andrews JPM, Doris MK, Shah ASV, BouHaidar R, Alcaide-Corral CJ, Williams MC, Leipsic JA, Dweck MR, MacRae VE, Newby DE, Tavares AAS, Sellers SL (2020). Ex vivo 18F-fluoride uptake and hydroxyapatite deposition in human coronary atherosclerosis. Sci Reports.

[167] Nadra I, Boccaccini AR, Philippidis P, Whelan LC, McCarthy GM, Haskard DO, Landis RC (2008). Effect of particle size on hydroxyapatite crystal-induced tumor necrosis factor alpha secretion by macrophages. Atherosclerosis.

[168] Nadra I, Mason JC, Philippidis P, Florey O, Smythe CDW, McCarthy GM, Landis RC, Haskard DO (2005). Proinflammatory activation of macrophages by basic calcium phosphate crystals via protein kinase C and MAP kinase pathways. Circ Res.

[169] Nakahara T, Strauss HW (2017). From inflammation to calcification in atherosclerosis. Eur J Nucl Med Mol Imaging.

[170] New SEP, Aikawa E (2011). Molecular imaging insights into early inflammatory stages of arterial and aortic valve calcification. Circ Res.

[171] New SEP, Aikawa E (2013). The role of extracellular vesicles in de novo mineralization: an additional novel mechanism of cardiovascular calcification. Arterioscler Thromb Vasc Biol.

[172] Newby AC (2006). Do metalloproteinases destabilize vulnerable atherosclerotic plaques?. Curr Opin Lipidol.

[173] Newby AC (2008). Metalloproteinase expression in monocytes and macrophages and its relationship to atherosclerotic plaque instability. Arterioscler Thromb Vasc Biol.

[174] Newby AC (2012). Matrix metalloproteinase inhibition therapy for vascular diseases. Vascul Pharmacol.

[175] Newby AC (2016). Metalloproteinase production from macrophages – a perfect storm leading to atherosclerotic plaque rupture and myocardial infarction. Exp Physiol.

[176] Newby DE, Son PDA, Berry C, Boon NA, Dweck MR, Flather M, Forbes J, Hunter A, Lewis S, MacLean S, Mills NL, John Norrie MS, Giles Roditi MD, Shah ASV, Timmis AD, van Beek EJR, Williams MC (2018). Coronary CT angiography and 5-year risk of myocardial infarction. N Engl J Med.

[177] Ngai D, Lino M, Bendeck MP (2018). Cell-matrix interactions and matricrine signaling in the pathogenesis of vascular calcification. Front Cardiovasc Med.

[178] Ngai D, Lino M, Rothenberg KE, Simmons CA, Fernandez-Gonzalez R, Bendeck MP (2020). DDR1 (Discoidin Domain Receptor-1)-RhoA (Ras Homolog Family Member A) axis senses matrix stiffness to promote vascular calcification. Arterioscler Thromb Vasc Biol.

[179] Nowak WN, Deng J, Ruan XZ, Xu Q (2017). Reactive oxygen species generation and atherosclerosis. Arterioscler Thromb Vasc Biol.

[180] O’Brien ER, Garvin MR, Stewart DK, Hinohara T, Simpson JB, Schwartz SM, Giachelli CM (1994). Osteopontin is synthesized by macrophage, smooth muscle, and endothelial cells in primary and restenotic human coronary atherosclerotic plaques. Arterioscler Thromb A J Vasc Biol.

[181] Ohayon J, Finet G, Gharib AM, Herzka DA, Tracqui P, Heroux J, Rioufol G, Kotys MS, Elagha A, Pettigrew RI (2008). Necrotic core thickness and positive arterial remodeling index: emergent biomechanical factors for evaluating the risk of plaque rupture. Am J Physiol Hear Circ Physiol.

[182] Olejarz W, Łacheta D, Kubiak-Tomaszewska G (2020). Matrix metalloproteinases as biomarkers of atherosclerotic plaque instability. Int J Mol Sci.

[183] Otsuka F, Sakakura K, Yahagi K, Joner M, Virmani R (2014). Has our understanding of calcification in human coronary atherosclerosis progressed?. Arterioscler Thromb Vasc Biol.

[184] Ovchinnikova O, Robertson AKL, Wågsäter D, Folco EJ, Hyry M, Myllyharju J, Eriksson P, Libby P, Hansson GK (2009). T-cell activation leads to reduced collagen maturation in atherosclerotic plaques of Apoe-/- mice. Am J Pathol.

[185] Ovchinnikova OA, Folkersen L, Persson J, Lindeman JHN, Ueland T, Aukrust P, Gavrisheva N, Shlyakhto E, Paulsson-Berne G, Hedin U, Olofsson PS, Hansson GK (2014). The collagen cross-linking enzyme lysyl oxidase is associated with the healing of human atherosclerotic lesions. J Intern Med.

[186] Oviedo-Orta E, Bermudez-Fajardo A, Karanam S, Benbow U, Newby AC (2008). Comparison of MMP-2 and MMP-9 secretion from T helper 0, 1 and 2 lymphocytes alone and in coculture with macrophages. Immunology.

[187] Panwar P, Du X, Sharma V, Lamour G, Castro M, Li H, Brömme D (2013). Effects of cysteine proteases on the structural and mechanical properties of collagen fibers. J Biol Chem.

[188] Parameswaran N, Patial S (2010). Tumor necrosis factor-α signaling in macrophages. Crit Rev Eukaryot Gene Expr.

[189] Patterson ML, Atkinson SJ, Knäuper V, Murphy G (2001). Specific collagenolysis by gelatinase A, MMP-2, is determined by the hemopexin domain and not the fibronectin-like domain. FEBS Lett.

[190] Pazár B, Ea H-K, Narayan S, Kolly L, Bagnoud N, Chobaz V, Roger T, Lioté F, So A, Busso N (2011). Basic calcium phosphate crystals induce monocyte/macrophage IL-1β secretion through the NLRP3 inflammasome in vitro. J Immunol.

[191] Perrotta I, Perri E (2017). Ultrastructural, elemental and mineralogical analysis of vascular calcification in atherosclerosis. Microsc Microanal.

[192] Persson IM, Pettersson NF, Liu J, Håkansson HF, Örbom A, Zandt RI, Gidlöf R, Sydoff M, von Wachenfeldt K, Olsson LE (2020). Longitudinal imaging using pet/ct with collagen-i pet-tracer and mri for assessment of fibrotic and inflammatory lesions in a rat lung injury model. J Clin Med.

[193] Plenz GAM, Deng MC, Robenek H, Völker W (2003). Vascular collagens: spotlight on the role of type VIII collagen in atherogenesis. Atherosclerosis.

[194] Polonskaya YV, Kashtanova EV, Murashov IS, Striukova EV, Kurguzov AV, Stakhneva EM, Shramko VS, Maslatsov NA, Chernyavsky AM, Ragino YI (2021). Association of matrix metalloproteinases with coronary artery calcification in patients with CHD. J Pers Med.

[195] Poulsen CB, Mehta VV, Holm NR, Pareek N, Post AL, Kilic ID, Banya WAS, Dall’Ara G, Mattesini A, Bjørklund MM, Andersen NP, Grøndal AK, Petretto E, Foin N, Davies JE, Di Mario C, Bentzon JF, Bøtker HE, Falk E, Krams R, De Silva R (2015). Inducing persistent flow disturbances accelerates atherogenesis and promotes thin cap fibroatheroma development in D374Y-PCSK9 hypercholesterolemic minipigs. Circulation.

[196] Proudfoot D, Skepper JN, Hegyi L, Farzaneh-Far A, Shanahan CM, Weissberg PL (2001). The role of apoptosis in the initiation of vascular calcification. Zeitschrift für Kardiol.

[197] Purroy A, Roncal C, Orbe J, Meilhac O, Belzunce M, Zalba G, Villa-Bellosta R, Andrés V, Parks WC, Páramo JA, Rodríguez JA (2018). Matrix metalloproteinase-10 deficiency delays atherosclerosis progression and plaque calcification. Atherosclerosis.

[198] Puxkandl R, Zizak I, Paris O, Keckes J, Tesch W, Bernstorff S, Purslow P, Fratzl P (2002). Viscoelastic properties of collagen: synchrotron radiation investigations and structural model. Philos Trans R Soc B Biol Sci.

[199] Rajagopalan S, Meng XP, Ramasamy S, Harrison DG, Galis ZS (1996). Reactive oxygen species produced by macrophage-derived foam cells regulate the activity of vascular matrix metalloproteinases in vitro. Implications for atherosclerotic plaque stability. J Clin Invest.

[200] Rambhia SH, Liang X, Xenos M, Alemu Y, Maldonado N, Kelly A, Chakraborti S, Weinbaum S, Cardoso L, Einav S, Bluestein D (2012). Microcalcifications increase coronary vulnerable plaque rupture potential: a patient-based micro-ct fluid-structure interaction study. Ann Biomed Eng.

[201] Redgrave JN, Gallagher P, Lovett JK, Rothwell PM (2008). Critical cap thickness and rupture in symptomatic carotid plaques: the oxford plaque study. Stroke.

[202] Redondo S, Ruiz E, Padilla E, Gordillo-Moscoso A, Tejerina T (2007). Role of TGF-β1 in vascular smooth muscle cell apoptosis induced by Angiotensin II. Eur J Pharmacol.

[203] Rekhter MD (1999). Collagen synthesis in atherosclerosis: too much and not enough. Cardiovasc Res..

[204] Richardson PD, Davies MJ, Born GVR (1989). Influence of plaque configuration and stress distribution on fissuring of coronary atherosclerotic plaques. Lancet.

[205] Roijers RB, Debernardi N, Cleutjens JPM, Schurgers LJ, Mutsaers PHA, Van Der Vusse GJ (2011). Microcalcifications in early intimal lesions of atherosclerotic human coronary arteries. Am J Pathol.

[206] Ross R, Masuda J, Raines EW, Gown AM, Katsuda S, Sasahara M, Malden LT, Masuko H, Sato H (1990). Localization of PDGF-B protein in macrophages in all phases of atherogenesis. Science (80–).

[207] Rubbens MP, Mol A, Boerboom RA, Bank RA, Baaijens FPT, Bouten CVC (2009). Intermittent straining accelerates the development of tissue properties in engineered heart valve tissue. Tissue Eng Part A.

[208] Ruiz-Ortega M, Rodríguez-Vita J, Sanchez-Lopez E, Carvajal G, Egido J (2007). TGF-β signaling in vascular fibrosis. Cardiovasc Res.

[209] Ruiz JL, Weinbaum S, Aikawa E, Hutcheson JD (2016). Zooming in on the genesis of atherosclerotic plaque microcalcifications. J Physiol.

[210] Saito M, Marumo K (2010). Collagen cross-links as a determinant of bone quality: a possible explanation for bone fragility in aging, osteoporosis, and diabetes mellitus. Osteoporos Int.

[211] Sakamoto A, Kawakami R, Mori M, Guo L, Paek KH, Mosquera JV, Cornelissen A, Ghosh SKB, Kawai K, Konishi T, Fernandez R, Fuller DT, Xu W, Vozenilek AE, Sato Y, Jinnouchi H, Torii S, Turner AW, Akahori H, Kuntz S, Weinkauf CC, Lee PJ, Kutys R, Harris K, Killey AL, Mayhew CM, Ellis M, Weinstein LM, Gadhoke NV, Dhingra R, Ullman J, Dikongue A, Romero ME, Kolodgie FD, Miller CL, Virmani R, Finn AV (2023). CD163+ macrophages restrain vascular calcification, promoting the development of high-risk plaque. JCI Insight.

[212] Schaar JA, De Korte CL, Mastik F, Strijder C, Pasterkamp G, Boersma E, Serruys PW, Van Der Steen AFW (2003). Characterizing vulnerable plaque features with intravascular elastography. Circulation.

[213] Schlieper G, Aretz A, Verberckmoes SC, Krüger T, Behets GJ, Ghadimi R, Weirich TE, Rohrmann D, Langer S, Tordoir JH, Amann K, Westenfeld R, Brandenburg VM, D’Haese PC, Mayer J, Ketteler M, McKee MD, Floege JR (2010). Ultrastructural analysis of vascular calcifications in uremia. J Am Soc Nephrol.

[214] Schmid F, Sommer G, Rappolt M, Schulze-Bauer CAJ, Regitnig P, Holzapfel GA, Laggner P, Amenitsch H (2005). In situ tensile testing of human aortas by time-resolved small-angle X-ray scattering. J Synchrotron Radiat.

[215] Schnoor M, Cullen P, Lorkowski J, Stolle K, Robenek H, Troyer D, Rauterberg J, Lorkowski S (2008). Production of type VI collagen by human macrophages: a new dimension in macrophage functional heterogeneity. J Immunol.

[216] Schoenborn S, Pirola S, Woodruff MA, Allenby MC (2022). Fluid-structure interaction within models of patient-specific arteries: computational simulations and experimental validations. IEEE Rev Biomed Eng.

[217] Schrijvers DM, De Meyer GRY, Herman AG, Martinet W (2007). Phagocytosis in atherosclerosis: molecular mechanisms and implications for plaque progression and stability. Cardiovasc Res.

[218] Shekhonin BV, Domogatsky SP, Idelson GL, Koteliansky VE, Rukosuev VS (1987). Relative distribution of fibronectin and type I, III, IV, V collagens in normal and atherosclerotic intima of human arteries. Atherosclerosis.

[219] Shioi A, Katagi M, Okuno Y, Mori K, Jono S, Koyama H, Nishizawa Y (2002). Induction of bone-type alkaline phosphatase in human vascular smooth muscle cells: roles of tumor necrosis factor-α and oncostatin M derived from macrophages. Circ Res.

[220] Simionescu A, Philips K, Vyavahare N (2005). Elastin-derived peptides and TGF-β1 induce osteogenic responses in smooth muscle cells. Biochem Biophys Res Commun.

[221] Simionescu A, Simionescu DT, Vyavahare NR (2007). Osteogenic responses in fibroblasts activated by elastin degradation products and transforming growth factor-β1: role of myofibroblasts in vascular calcification. Am J Pathol.

[222] Singh D, Rai V, Agrawal KD (2023). Regulation of collagen i and collagen III in tissue injury and regeneration. Cardiol Cardiovasc Med.

[223] Slager CJ, Wentzell JJ, Gijsen FJH, Thury A, van der Waal AC, Schaar JA, Serruys PW (2005). The role of shear stress in the destabilization of vulnerable plaques and related therapeutic implications. Nat Clin Pract Cardiovasc Med.

[224] Snedeker JG, Gautieri A (2014). The role of collagen crosslinks in ageing and diabetes—the good, the bad, and the ugly. Muscles Ligaments Tendons J.

[225] Song B, Bie Y, Feng H, Xie B, Liu M, Zhao F (2022). Inflammatory factors driving atherosclerotic plaque progression new insights. J Transl Intern Med.

[226] Song YL, Ford JW, Gordon D, Shanley CJ (2000). Regulation of lysyl oxidase by interferon-y in rat aortic smooth muscle cells. Arter Thromb Vasc Biol.

[227] Sridharan R, Genoud KJ, Kelly DJ, O’Brien FJ (2020). Hydroxyapatite particle shape and size influence MSC osteogenesis by directing the macrophage phenotype in collagen-hydroxyapatite scaffolds. ACS Appl Bio Mater.

[228] Stöger JL, Gijbels MJJ, Van Der VS, Manca M, Van Der LCM, Biessen EAL, Daemen MJAP, Lutgens E, De WMPJ (2012). Distribution of macrophage polarization markers in human atherosclerosis. Atherosclerosis.

[229] Sukhova GK, Shi GP, Simon DI, Chapman HA, Libby P (1998). Expression of the elastolytic cathepsins S and K in human atheroma and regulation of their production in smooth muscle cells. J Clin Invest.

[230] Wess TJ, Fratzl P (2008). Collagen fibrillar structure and hierarchies. Collagen structure and mechanics.

[231] Tabas I, KaE B (2016). Macrophage phenotype and function in different stages of atherosclerosis. Circ Res.

[232] Tang D, Yang C, Huang S, Mani V, Zheng J, Woodard PK, Robson P, Teng Z, Dweck M, Fayad ZA (2017). Cap inflammation leads to higher plaque cap strain and lower cap stress: an MRI-PET/CT-based FSI modeling approach. J Biomech.

[233] Tang Y, Huang M, Zheng W, Robson T, Dweck F (2017). Cap inflammation leads to higher plaque cap strain and lower cap stress: an MRI-PET/CT-based FSI modeling approach. J Biomech.

[234] Teng Z, Feng J, Zhang Y, Sutcliffe MPF, Huang Y, Brown AJ, Jing Z, Lu Q, Gillard JH (2015). A uni-extension study on the ultimate material strength and extreme extensibility of atherosclerotic tissue in human carotid plaques. J Biomech.

[235] Tesauro M, Mauriello A, Rovella V, Annicchiarico-Petruzzelli M, Cardillo C, Melino G, Di Daniele N (2017). Arterial ageing: from endothelial dysfunction to vascular calcification. J Intern Med.

[236] Thorp E, Tabas I (2009). Mechanisms and consequences of efferocytosis in advanced atherosclerosis. J Leukoc Biol.

[237] Toma I, McCaffrey TA (2012). Transforming growth factor-β and atherosclerosis: interwoven atherogenic and atheroprotective aspects. Physiol Behav.

[238] Tornifoglio B, Johnston RD, Stone AJ, Kerskens C, Lally C (2023). Microstructural and mechanical insight into atherosclerotic plaques: an ex vivo DTI study to better assess plaque vulnerability. Biomech Model Mechanobiol.

[239] Tornifoglio B, Stone AJ, Kerskens C, Lally C (2022). Ex vivo study using diffusion tensor imaging to identify biomarkers of atherosclerotic disease in human cadaveric carotid arteries. Arterioscler Thromb Vasc Biol.

[240] Tornifoglio B (2022) An investigation into diffusion tensor imaging-derived metrics in arterial tissue as biomarkers for disease progression, plaque rupture and graft recellularisation

[241] Torun SG, MunozCrielaard PMH, Verhagen HJM, Kremers GJ, van der Steen AFW, Akyildiz AC (2023). Local characterization of collagen architecture and mechanical failure properties of fibrous plaque tissue of atherosclerotic human carotid arteries. Acta Biomater.

[242] Del Turco S, Basta G (2012). An update on advanced glycation endproducts and atherosclerosis. BioFactors.

[243] Tyson J, Bundy K, Roach C, Douglas H, Ventura V, Segars MF, Schwartz O, Simpson CL (2020). Mechanisms of the osteogenic switch of smooth muscle cells in vascular calcification: Wnt signaling, bmps, mechanotransduction, and endmt. Bioengineering.

[244] Vasse GF, Russo S, Barcaru A, Oun AAA, Dolga AM, Van RP, Kwiatkowski M, Govorukhina N, Bischoff R, Melgert BN (2023). Collagen type I alters the proteomic signature of macrophages in a collagen morphology—dependent manner. Sci Rep.

[245] Vengrenyuk Y, Cardoso L, Weinbaum S (2008). Micro-CT based analysis of a new paradigm for vulnerable plaque rupture: cellular microcalcifications in fibrous caps. MCB Mol Cell Biomech.

[246] Vengrenyuk Y, Carlier S, Xanthos S, Cardoso L, Ganatos P, Virmnani R, Einav S, Gilchrist L, Weinbaum S (2006). A hypothesis for vulnerable plaque rupture due to stress-induced debonding around cellular microcalcifications in thin fibrous caps. Proc Natl Acad Sci USA.

[247] Vidak E, Javoršek U, Vizovišek M, Turk B (2019). Cysteine cathepsins and their extracellular roles: shaping the microenvironment. Cells.

[248] Villa-Bellosta R, Hamczyk MR, André V (2017). Novel phosphate-activated macrophages prevent ectopic calcification by increasing extracellular ATP and pyrophosphate. PLoS ONE.

[249] Virmani R, Burke AP, Farb A, Kolodgie FD (2006). Pathology of the vulnerable plaque. J Am Coll Cardiol.

[250] Voss B, Rauterberg J (1986). Localization of collagen types I, III, IV and V, fibronectin and laminin in human arteries by the indirect immunofluorescence method. Pathol Res Pract.

[251] Van Der Wal AC, Becker AE, Van Der Loos CM, Das PK (1994). Site of intimal rupture or erosion of thrombosed coronary atherosclerotic plaques is characterized by an inflammatory process irrespective of the dominant plaque morphology. Circulation.

[252] Wang J, Uryga AK, Reinhold J, Figg N, Baker L, Finigan A, Gray K, Kumar S, Clarke M, Bennett M (2015). Vascular smooth muscle cell senescence promotes atherosclerosis and features of plaque vulnerability. Circulation.

[253] Wang S, Jiang H, Zheng C, Gu M, Zheng X (2022). Secretion of BMP-2 by tumor-associated macrophages (TAM) promotes microcalcifications in breast cancer. BMC Cancer.

[254] Wang Y, Osborne MT, Tung B, Li M, Li Y (2018). Imaging cardiovascular calcification. J Am Heart Assoc.

[255] Watson KE, Parhami F, Shin V, Demer LL (1998). Fibronectin and collagen I matrixes promote calcification of vascular cells in vitro, whereas collagen IV matrix is inhibitory. Arterioscler Thromb Vasc Biol.

[256] Weitkamp B, Cullen P, Plenz G, Robenek H, Rauterberg J (1999). Human macrophages synthesize type VIII collagen in vitro and in the atherosclerotic plaque. FASEB J.

[257] Wenk JF, Papadopoulos P, Zohdi TI (2010). Numerical modeling of stress in stenotic arteries with microcalcifications: a micromechanical approximation. J Biomech Eng.

[258] Wesley RB, Meng X, Godin D, Galis ZS (1998). Extracellular matrix modulates macrophage functions characteristic to atheroma: collagen type I enhances acquisition of resident macrophage traits by human peripheral blood monocytes in vitro. Arterioscler Thromb Vasc Biol.

[259] Williams MC, Moss AJ, Dweck M, Adamson PD, Alam S, Hunter A, Shah ASV, Pawade T, Weir-McCall JR, Roditi G, van Beek EJR, Newby DE, Nicol ED (2019). Coronary artery plaque characteristics associated with adverse outcomes in the SCOT-HEART study. J Am Coll Cardiol.

[260] Wissing TB, Van Haaften EE, Koch SE, Ippel BD, Kurniawan NA, Bouten CVC, Smits AIPM (2020). Hemodynamic loads distinctively impact the secretory profile of biomaterial-activated macrophages-implications for in situ vascular tissue engineering. Biomater Sci.

[261] Wissing TB, Van der Heiden K, Serra SM, Smits AIPM, Bouten CVC, Gijsen FJH (2022). Tissue-engineered collagenous fibrous cap models to systematically elucidate atherosclerotic plaque rupture. Sci Rep.

[262] Witherel CE, Abebayehu D, Barker TH, Spiller KL (2019). Macrophage and fibroblast interactions in biomaterial-mediated fibrosis. Adv Healthc Mater.

[263] Wolf D, Ley K (2019). Immunity and inflammation in atherosclerosis. Circ Res.

[264] Wong KKL, Thavornpattanapong P, Cheung SCP, Sun Z, Tu J (2012). Effect of calcification on the mechanical stability of plaque based on a three-dimensional carotid bifurcation model. BMC Cardiovasc Disord.

[265] Wong KKL, Wu J, Liu G, Huang W, Ghista DN (2020). Coronary arteries hemodynamics: effect of arterial geometry on hemodynamic parameters causing atherosclerosis. Med Biol Eng Comput.

[266] Wu H, Du Q, Dai Q, Ge J, Cheng X (2018). Cysteine protease cathepsins in atherosclerotic cardiovascular diseases. J Atheroscler Thromb.

[267] Xiao L, Shiwaku Y, Hamai R, Tsuchiya K, Sasaki K, Suzuki O (2021). Macrophage polarization related to crystal phases of calcium phosphate biomaterials. Int J Mol Sci.

[268] Yahagi K, Kolodgie FD, Otsuka F, Finn AV, Davis HR, Joner M, Virmani R (2016). Pathophysiology of native coronary, vein graft, and in-stent atherosclerosis. Nat Rev Cardiol.

[269] Yurdagul A (2022). Crosstalk between macrophages and vascular smooth muscle cells in atherosclerotic plaque stability. Arterioscler Thromb Vasc Biol.

[270] Zazzeroni L, Faggioli G, Pasquinelli G (2018). Mechanisms of arterial calcification: the role of matrix vesicles. Eur J Vasc Endovasc Surg.

